# Supplementary material for: SONAR enables cell type deconvolution with spatially weighted Poisson-Gamma model for spatial transcriptomics
Source: Nat Commun. 2023 Aug 7;14:4727. doi: 10.1038/s41467-023-40458-9 (PMC10406862; doi:10.1038/s41467-023-40458-9)
Supplement: Supplementary file 1 — Supplementary Information [file 41467_2023_40458_MOESM1_ESM.pdf]

SONAR enables cell type deconvolution with spatially weighted Poisson-Gamma model for spatial transcriptomics

Zhiyuan Liu[1,2], Dafei Wu[1], Weiwei Zhai *[1,2,3] and Liang Ma †[1]

[1]Key Laboratory of Zoological Systematics and Evolution, Institute of Zoology, Chinese Academy of Sciences, Beijing, 100101, China
[2]University of the Chinese Academy of Sciences, Beijing 100049, China
[3]Center for Excellence in Animal Evolution and Genetics, Chinese Academy of Sciences, Kunming, 650223, China

Supplementary Note

Supplementary Note 1: details in likelihood function

In the Methods section, we model the expected expression rate, $\lambda_{i,g}$, of gene $g \in \mathcal{G}$ at spot $i \in \mathcal{I}$ as a linear function of reference cell type signatures $s_{t,g}, \forall t \in \mathcal{T}$:

$$\lambda_{i,g} = \beta_{i,0} + \sum_{t=1}^{T} \beta_{i,t} \cdot s_{t,g}, \tag{1}$$

where the intercept $\beta_{i,0}$ defines the spot-specific effect, and $\beta_{i,t}$s represent the mixture weights of reference cell types $t \in \mathcal{T}$. For convenience, we refer all the unknown parameters to be estimated in the above linear model for spot i as $\boldsymbol{\beta}_i = \{\beta_{i,j} : j \in \mathcal{T} \cup \{0\}\}$.

For each spot $i \in \mathcal{I}$ and gene $g \in \mathcal{G}$, we model y_{ig} by a Poisson-Gamma distribution as:

$$y_{i,g}|\lambda_{i,g}, V_{i,g} \sim \text{Poisson}(\Phi_i \lambda_{i,g} V_{i,g}), \tag{2}$$

where Φ_i is the total number of UMIs in spot i, which serves as scale factor for sequencing depth at each spatial location; and $V_{i,g} \sim \text{Gamma}(\alpha_i, \alpha_i)$ is the unobserved multiplicative random effect of heterogeneity, which accounts for gene- and spatial-specific over-dispersion. With formula (1) and distribution (2), we can write the posterior distribution density of $y_{i,g}$ as:

$$\begin{aligned} P(y_{i,g}|\boldsymbol{\beta}_i, \alpha_i) &= \frac{(\Phi_i \cdot \lambda_{i,g} \cdot V_{i,g})^{y_{i,g}}}{y_{i,g}!} \cdot e^{-\Phi_i \cdot \lambda_{i,g} \cdot V_{i,g}} \\ &=: f_{i,g}(\boldsymbol{\beta}_i, V_{i,g}) \end{aligned} \tag{3}$$

Since the random parameter $V_{i,g}$ is not observable, and the density function of it is:

$$g(V_{i,g}) = \frac{V_{i,g}^{\alpha_i - 1} \cdot e^{-V_{i,g} \cdot \alpha_i} \cdot {\alpha_i}^{\alpha_i}}{\Gamma(\alpha_i)} \tag{4}$$

*Corresponding author: weiweizhai@ioz.ac.cn
†Corresponding author: maliang@ioz.ac.cn

According to the formula (3) and (4), we can obtain the marginal density of $y_{i,g}$ by integrating out $V_{i,g}$:

$$\begin{aligned} P(y_{i,g}|\boldsymbol{\beta}_i, \alpha_i) &= \int_0^{\infty} f_{i,g}(\boldsymbol{\beta}_i, V_{i,g}) \cdot g(V_{i,g})\ dV_{i,g} \\ &= \frac{\Gamma\left(\alpha_i + y_{i,g}\right)}{\Gamma\left(\alpha_i\right)\Gamma(y_{i,g}+1)} \left(\frac{\alpha_i}{\alpha_i + \Phi_i \cdot \lambda_{i,g}}\right)^{\alpha_i} \left(\frac{\Phi_i \cdot \lambda_{i,g}}{\Phi_i \cdot \lambda_{i,g} + \alpha_i}\right)^{y_{i,g}} \end{aligned}$$

Supplementary Note 2: Details for evaluation on different spot scale

We investigated the impact of different resolution and spot scale using human heart spatial data (obtained by in situ sequencing), which containing 17,444 spots/cells. We originally gridded the data into pseudo-spots of size 454*424 pixels, which containing on average 16.5 cells. To assess the effect of spot scale, we further gridded the data with reduced sizes corresponding to 75% (356*318 pixels, with an average of 10 cells) and 50% (227*212 pixels, with an average of 4.9 cells) of the original grid size. We evaluated the Pearson correlation (PCC) between the predicted proportion and the ground truth of each cell type over all pseudo-spots. SONAR maintained its advantage over the other compared methods, although there is a decrease in performance from the original grid size to the higher resolution size (Supplementary Fig. 10a). RCTD did not produce a result in the 50%-sized data due to an insufficient number of features (71.5 UMI counts per spot), but had comparable performance to SONAR on the other two datasets. In addition, we further evaluate the distribution of cNCCs and SPCs type, which were previously found to be predominantly distributed in the outflow tract region (OFT). In all resolutions tested, SONAR is the only method that can successfully recover the localization of these cell types (Supplementary Fig. 10b, c). SONAR also shows the highest PCCs between the estimated proportion of cNCCs and SPCs and the truth (Supplementary Fig. 10b).

Supplementary Note 3: Computational time of comparing algorithms.

To show the computation efficiency of SONAR, we recorded the computational time of SONAR and other compared methods on a simulation datasets, a Visium dataset (human liver HCC) and a large-scale Slide-seqV2 dataset (mouse hippocampus). The simulation dataset consists of 800 spots and 2845 genes, the Visium dataset has 2,791 spots and 17,735 genes and the Slide-seqV2 dataset has 41795 spots and 5093 genes. Computation of SONAR, RCTD, CARD, SPOTlight and SpatialDWLS were on the CPU processor: Intel(R) Xeon(R) Gold 6248R CPU @ 3.00GHz processor. Computation of Cell2location, Stereoscope and SD2 were performed on a RTX 3090 GPU processor. The results show that SONAR has a high computational efficiency in all tested datasets (Supplementary Fig. 32).

Supplementary Note 4: Selection of bandwidth

In the kernel function, the bandwidth is the farthest neighbor that can be utilized and acts as a coefficient to normalize the distance. The larger the bandwidth, the more distant neighbors will be used, but the more distant neighbors may have less similarity with the target point, which may introduce unnecessary noise and increase the computational pressure of the algorithm. Therefore, it is necessary to choose an appropriate bandwidth.

The bandwidth determines the maximum radius of neighbors. In the original geographically weighted regression framework, the optimal bandwidth can be optimized through cross-validation or the Akaike information criterion (1).

We tested the effect of different bandwidth settings based on simulation data of Background and Block scenarios, which have complex patterns (Supplementary Fig. 3a bottom of Background/Block pattern). We found that even with a small bandwidth ($b = 1.2$), the performance of the algorithm

could be significantly improved by using neighbor information. Increasing bandwidth did not make an explicit improvement in performance but significantly increased computation time (Supplementary Fig. 33a).

Recent developments have proposed a multi-scale geographically weighted regression or MGWR, which allows different bandwidths for each individual variable (2). However, the current MGWR framework is limited to Gaussian models and is still computationally expensive. We further examined the effect of bandwidth on individual cell types using simulation data with a block pattern (Supplementary Fig. 3a bottom of Block Pattern). We find that, in most cell types, both versions of SONAR performed better with a small bandwidth, which only accounted for the neighbors in nearest proximal (first-order). The only cell type that tend to favor large bandwidth had the largest Moran's I value, which is as expected (Supplementary Table 7). However, setting a larger bandwidth tends to over-smooth the estimated parameter surface in all cell types (Supplementary Fig. 33b), especially those estimated by SONAR-0. Such overly smoothed estimates not only introduce greater amount of estimation bias, but also distort important local structures, such as region boundaries or spatially dispersed cell types (Supplementary Fig. 33b,c Type 2 and Fig. 2f). The extra steps of pre-clustering and elastic weighting in SONAR mitigate the errors caused by over smoothness.

Finally, we tested different bandwidth settings on mouse visual cortex data (3). The result also favors a small bandwidth in SONAR ($b = 1.2$, Supplementary Fig. 33d).

In sum, we choose to use 1.2 as the default bandwidth which accounts for the neighbors in nearest proximal (first-order neighbors).

Supplementary Note 5: Optimization method of solving likelihood function

For each spot i, we want to solve the MLE for non-negative parameters $\boldsymbol{\beta}_i$ and α_i in following optimization problem:

$$\begin{aligned} \underset{\boldsymbol{\beta}_i,\alpha_i}{\operatorname{argmax}}\, T(\boldsymbol{\beta}_i,\alpha_i) &= \sum_{n\in\mathcal{N}_i} L_n(\boldsymbol{\beta}_i,\alpha_i)\cdot w(d_{n,i}) \\ \text{s.t. } \boldsymbol{\beta}_i,\alpha_i &\geq 0 \end{aligned} \tag{5}$$

Here we write the parameters to be soloved as D dimensional vector $\boldsymbol{x} = (\boldsymbol{\beta}_i, \alpha_i)^T$, where D equals the concatenated dimension of $\boldsymbol{\beta}_i$ and α_i. The optimization problem (5) can thus be converted to a constraint multivariate nonlinear optimization problem:

$$\begin{aligned} \underset{\boldsymbol{x}}{\operatorname{argmax}}\, T(\boldsymbol{x}) &= \sum_{n\in\mathcal{N}_i} L_n(\boldsymbol{x})\cdot w(d_{n,i}) \\ \text{s.t. } \boldsymbol{x} &\geq 0 \end{aligned} \tag{6}$$

We solve the above problem with Interior Point algorithm in Algorithm 1:

Algorithm 1 Framework of Interior Point Algorithm

Input: Objective function: $-T(\boldsymbol{x})$ as defined before

Constraint: $\boldsymbol{x} \geq 0$

Initial point: $\boldsymbol{x^0} = (x_1^0, \cdots, x_D^0)$

Initial iteration: k:=1

Tolerance: $\epsilon \geq 0, \epsilon_1 \geq 0$

Barrier factor: μ^1

Output: Solution: $\boldsymbol{x}$

Set $B(\boldsymbol{x}, \mu) = -T(\boldsymbol{x}) - \mu \sum_{d=1}^{D} \ln x_d$

while $\left\|\nabla_x B(\boldsymbol{x}^{k-1}, \mu^k)\right\| > \epsilon_1$ **do**

$\boldsymbol{x}(\mu^k) = \operatorname{argmin}_{\boldsymbol{x}} B(\boldsymbol{x}, \mu^k)$

while $\mu^k \cdot \sum_{d=1}^{D} \ln(x_d(\mu^k)) > \epsilon$ **do**

$\boldsymbol{x}^{k+1} \leftarrow \boldsymbol{x}(\mu^k)$;

Choose $\mu^{k+1} < \mu^k$;

Set $k \leftarrow k + 1$;

Supplementary Note 6: Parameters for clustering

We performed a comparison of four clustering algorithms, K-means, Leiden, Louvain, SLM and applied different parameters (k = 3, 4, 5, 6 for K-means, resolution = 0.1, 0.4, 0.7, 1 for others) for each algorithm (Supplementary Fig. 34). We used the Kruskal-Wallis test to assess the differences among different conditions and found no significant difference. These results demonstrate the robustness of SONAR to the choice of pre-clustering algorithm and parameter. The robustness stems from the fact that pre-clustering only affects the selection of neighbor points, while the actual weights of each spot are determined by a combination of spatial kernel and elastic weighting, which makes the result more stable.

Supplementary Note 7: Details for simulation based on scRNA-seq data

In this section, we describe how we sample the cells from the PBMC scRNA-seq dataset according to the designed cell type profile for each spot. We generated the observed expression profile at each pseudo-spot by following these steps:

1. For each homogeneous region, we randomly selected several possible cell types (default 4 cell types) that could be present in this region.

2. We specified the expected distribution of each cell type in each region according to the designed scenarios of cell type composition and spatial patterns. We used Conway–Maxwell Poisson distribution with type specific expectation parameter to model the number of observed cells for each cell type in a pseudo-spot. We constrained each pseudo-spot to contains at most four cell types, including dominant types (default expectation parameter 6) and/or sparse types (default expectation 0.1).

3. For each pseudo-spot, we sampled cells from the annotated PBMC scRNA-seq dataset according to the expected distribution of each cell type. We pooled the gene expression counts of the sampled cells to obtain the expression profile of each pseudo-spot.

We divided our simulation into two schemes: Homo-Area and Compo-Area. In the first scheme, we design the cell type profile for each spot based on four local factors under a homogeneous region. The four local factors are:

- Abundance of dominant cell type: We varied the expected number of cells in dominant type from 8 to 2. Dominant types are cell types that have a high frequency and influence in a region.
- Abundance of sparse cell types: We changed the expected number of cells in sparse type from 0.3 to 0. Sparse types are cell types that have a low frequency and influence in a region.
- Number of dominant types: We changed the number of dominant cell types from 4 to 1.
- Relative proportion of multiple dominant types: We varied the expected number of cells in two dominant types from even expectations (4 cells each) to uneven expectations (8 and 0 cells).

In the second scheme, we design the cell type profile for each spot based on three general spatial structures under a composite region. We extended the spatial region to 20-by-40 spots size, and generated composite regions that consist of multiple homogeneous subregions. Homogeneous subregions are regions that have the same cell type composition at each spot. The three general spatial structures are:

- Transition mode: We designed three major modes of transition between two subregions: Jump-transition, Gradient-transition and Mixed-transition. Jump-transition means that there is no intermediate transition area between subregions, which forms a sharp and clear boundary. Gradient-transition refers to a soft boundary which forms a buffer area between subregions, where the cell type composition disperses in gradient from one side of subregion to the other side. Mix-transition also forms a soft boundary between subregions, but with cell type composition in the buffer area present in form of a uniform mixture over compositions of the two subregions.
- Spatial pattern: We designed three major patterns of subregions arrangement (with Jump-transition): Layer pattern, Block pattern, and Background pattern. Layer pattern refers to dividing the whole region into 2, 3, 4 or 5 layered subregions. Block pattern means that the whole area is divided into hierarchically blocked subregions. Background pattern represents one or more small subregions floating on a large background of homogeneous region.
- Abundance change: We generated two subregions with different expectations of dominant cells. The expectations ranged from 8 to 2 cells for each dominant type.

The homogeneous subregions were by default formed by one dominant cell type and three sparse types. We generated 5 replicates for each scenario of cell type composition with cell types sampled randomly.

Supplementary Figures

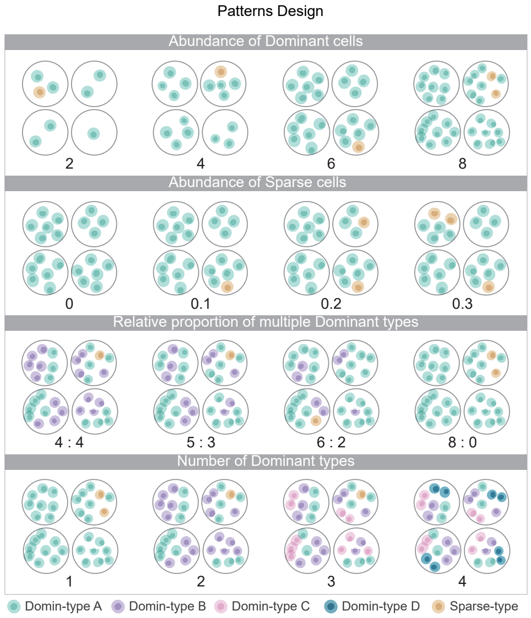

Supplementary Fig. 1 Patterns design in Homo-Area scheme.
Patterns design shown in the Homo-Area scheme. Each row represents 4 testing scenarios of 1 local factor. Different cell colors represent different cell types, and the grey circles represent the spots. In the first row, the abundance of dominant cells increases from left to right, and numbers indicate the expectation of dominant cell numbers within each spot. In the second row, the abundance of sparse cells increases, the expectation of sparse cell numbers within each spot increases from 0 to 0.3 per spot per type. In the third row, the ratio is composed of the expected cell numbers of two dominant types in one spot, from left to right, the ratio is from 4:4 to 8:0. In the forth row, from left to right, the number of dominant types per spot is 1 to 4.

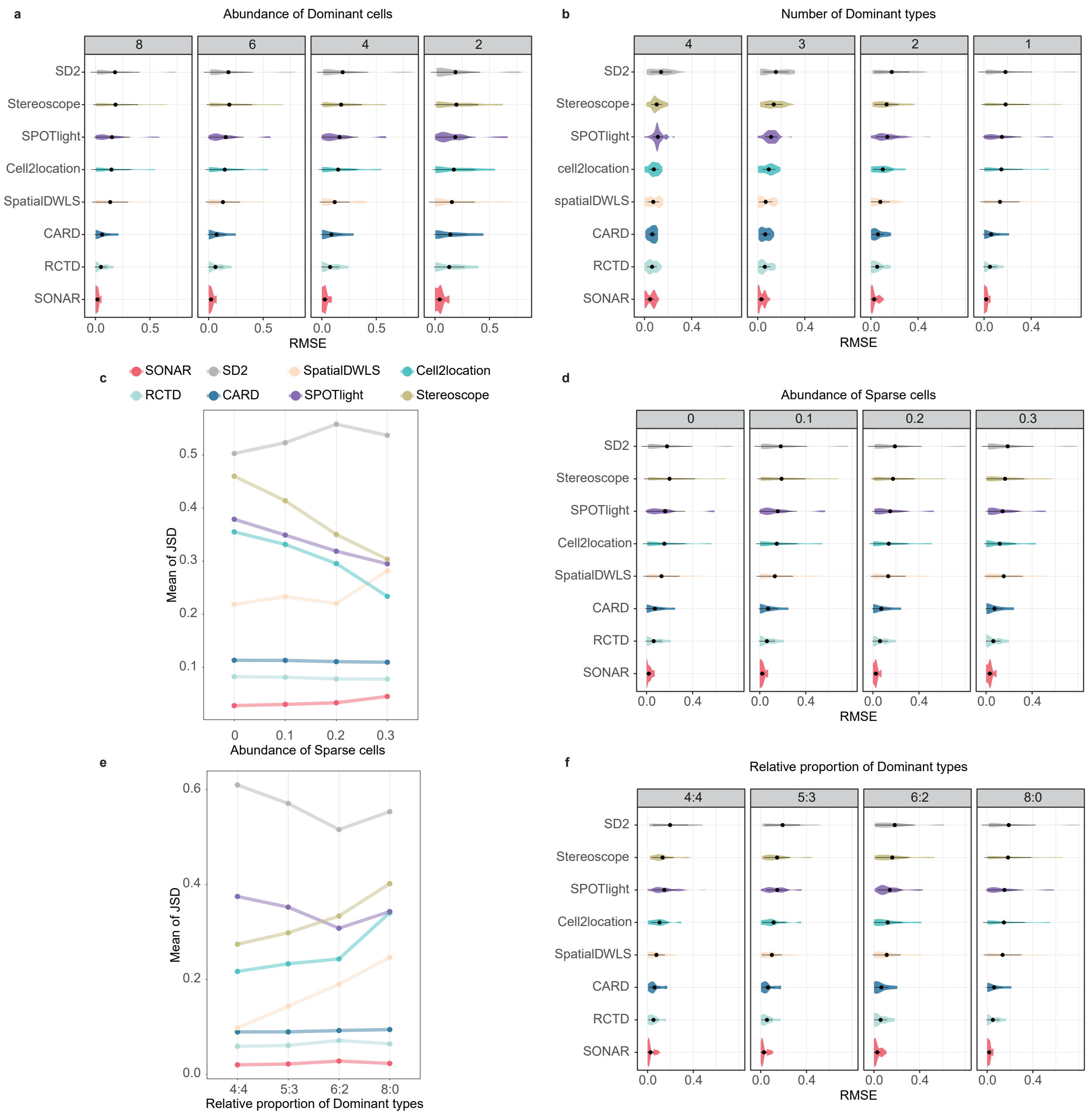

Supplementary Fig. 2 Benchmarking on Homo-Area scheme.
a, The violin plots show the RMSE for each type in the different dominant cells abundance testing scenarios (n = 40, 5 replicates ∗ 8 types) for all comparing algorithms. **b**, The violin plots show the RMSE for each type in the different dominant types number testing scenarios (n = 40, 5 replicates ∗ 8 types) for all comparing algorithms. **c**, The line plots show the JSD's mean of the testing scenarios on different sparse cells abundance (n = 2,000, 5 replicates ∗ 400 spots), and different colors represent different comparing algorithms. **d**, The violin plots show the RMSE for each type in the testing scenarios of different sparse cells abundance (n = 40, 5 replicates ∗ 8 types) for all comparing algorithms. **e**, The line plots show the JSD's mean of the testing scenarios on the relative proportion of two dominant types (n = 2,000, 5 replicates ∗ 400 spots), the color legend is same as **c**. **f**, The violin plots show the RMSE for each type in the testing scenarios on the relative proportion of two dominant types (n = 40, 5 replicates ∗ 8 types) for all comparing algorithms. In **a, b, d, f**,

the dot represents the mean of the violin plot, and the line represents mean ± sd. Source data are provided as a Source Data file.

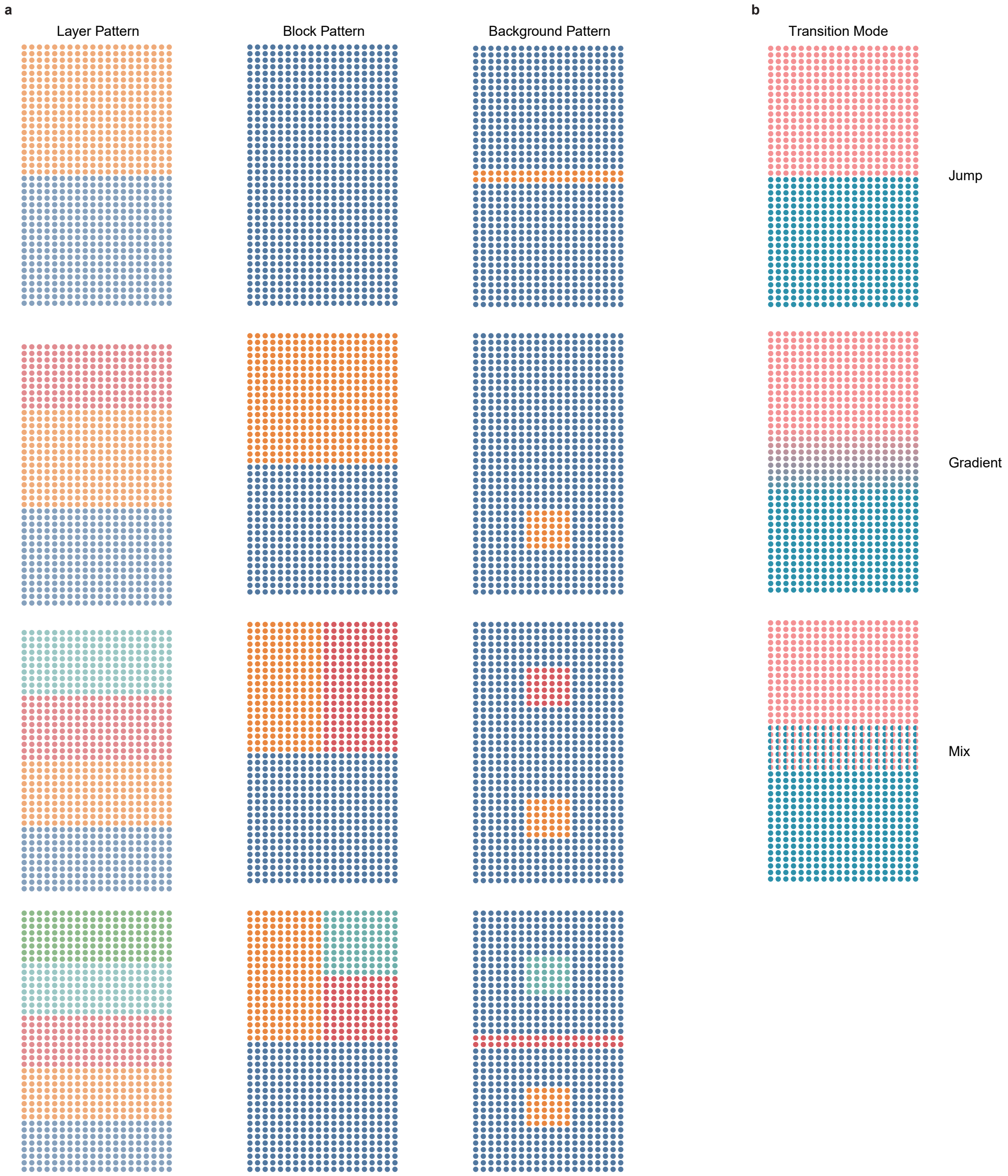

Supplementary Fig. 3 Patterns design in Compo-Area scheme.
a, Each column represents 4 types in the spatial pattern (Layer pattern, Block pattern, Background pattern) with increasing complexity from top to bottom. Different colors represent different subregions, and each dot represents a spot. **b**, Presentation of the three transition modes (Jump, Gradient, Mix), with different colors representing the characteristics of different subregions.

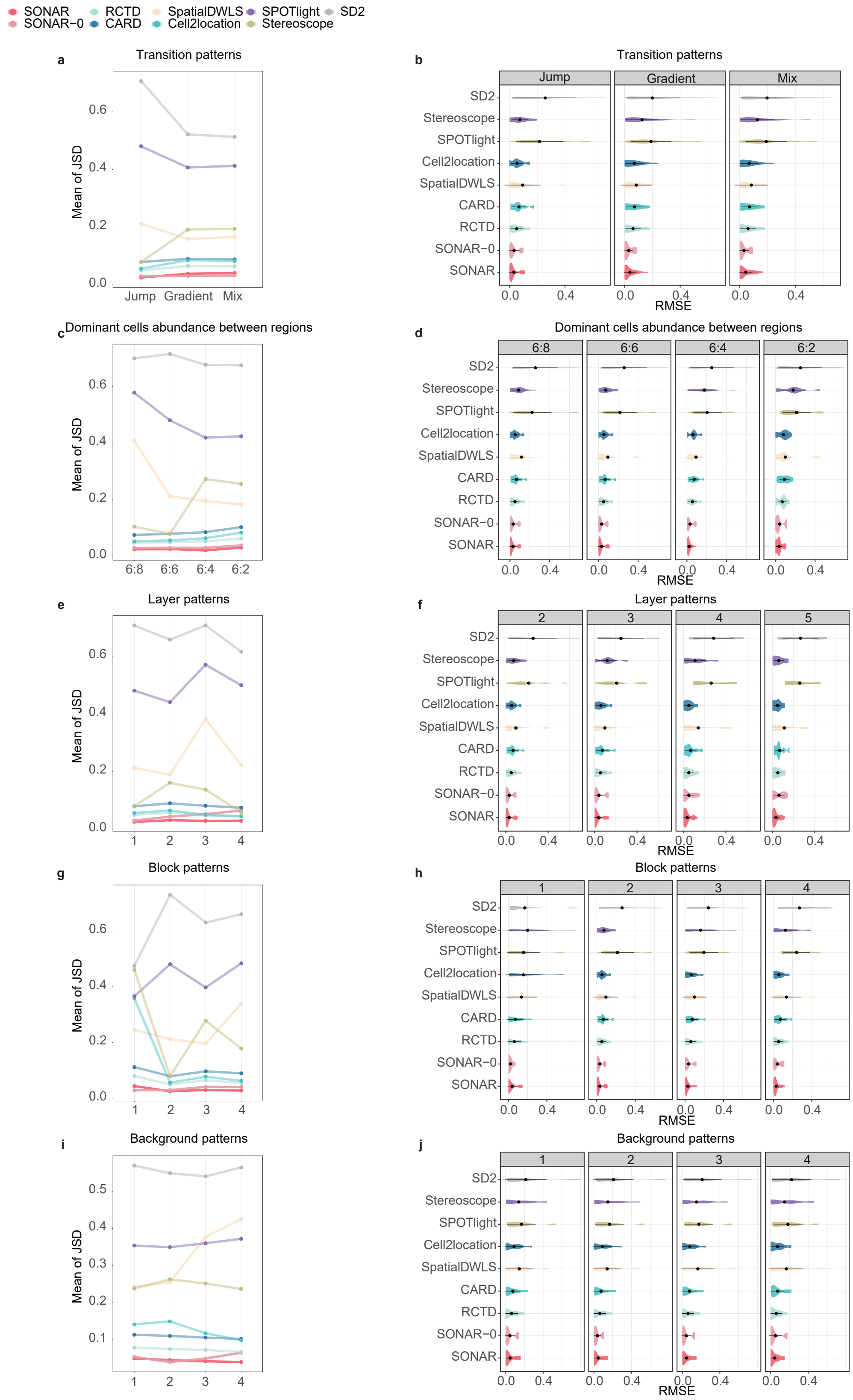
SONAR
SONAR−0
RCTD
CARD
SpatialDWLS
Cell2location
SPOTlight
Stereoscope
SD2
a
Transition patterns
Mean of JSD
Jump Gradient Mix
b
Transition patterns
Jump
Gradient
Mix
RMSE
c
Dominant cells abundance between regions
6:8 6:6 6:4 6:2
d
Dominant cells abundance between regions
e
Layer patterns
f
Layer patterns
g
Block patterns
h
Block patterns
i
Background patterns
j
Background patterns

Supplementary Fig. 4 Benchmarking on Compo-Area scheme.
a, The line plots show the JSD's mean of the testing scenarios on transition patterns (n = 4,000, 5 replicates ∗ 800 spots), and different colors represent different comparing algorithms. **b**, The violin plots show the RMSE for each type in the different transition patterns (n = 40, 5 replicates ∗ 8 types) for all comparing algorithms. **c**, The line plots show the JSD's mean of the testing scenarios on various dominant cells abundance between subregions (n = 4,000, 5 replicates ∗ 800 spots). **d**, The violin plots show the RMSE for each type on various dominant cells abundance between subregions (n = 40, 5 replicates ∗ 8 types) for all comparing algorithms. **e**, The line plots show the JSD's mean of the testing scenarios of layer patterns (n = 4,000, 5 replicates ∗ 800 spots), complexity increases from 1 to 4, corresponding to the top to bottom in the patterns design show (Supplementary Fig. 3). **f**, The violin plots show the RMSE for each type in the different layer patterns (n = 40, 5 replicates ∗ 8 types) for all comparing algorithms. **g**, The line plots show the JSD's mean of the testing scenarios of block patterns (n = 4,000, 5 replicates ∗ 800 spots), complexity increases from 1 to 4, corresponding to the top to bottom in the patterns design show (Supplementary Fig. 3). **h**, The violin plots show the RMSE for each type in the different block patterns (n = 40, 5 replicates ∗ 8 types) for all comparing algorithms. **i**, The line plots show the JSD's mean of the testing scenarios of background patterns (n = 4,000, 5 replicates ∗ 800 spots), complexity increases from 1 to 4, corresponding to the top to bottom in the patterns design show (Supplementary Fig. 3). **j**, The violin plots show the RMSE for each type in the different background patterns (n = 40, 5 replicates ∗ 8 types) for all comparing algorithms. In **b, d, f, h, j**, the dot represents the mean of the violin plot, and the line represents mean ± sd. Source data are provided as a Source Data file.

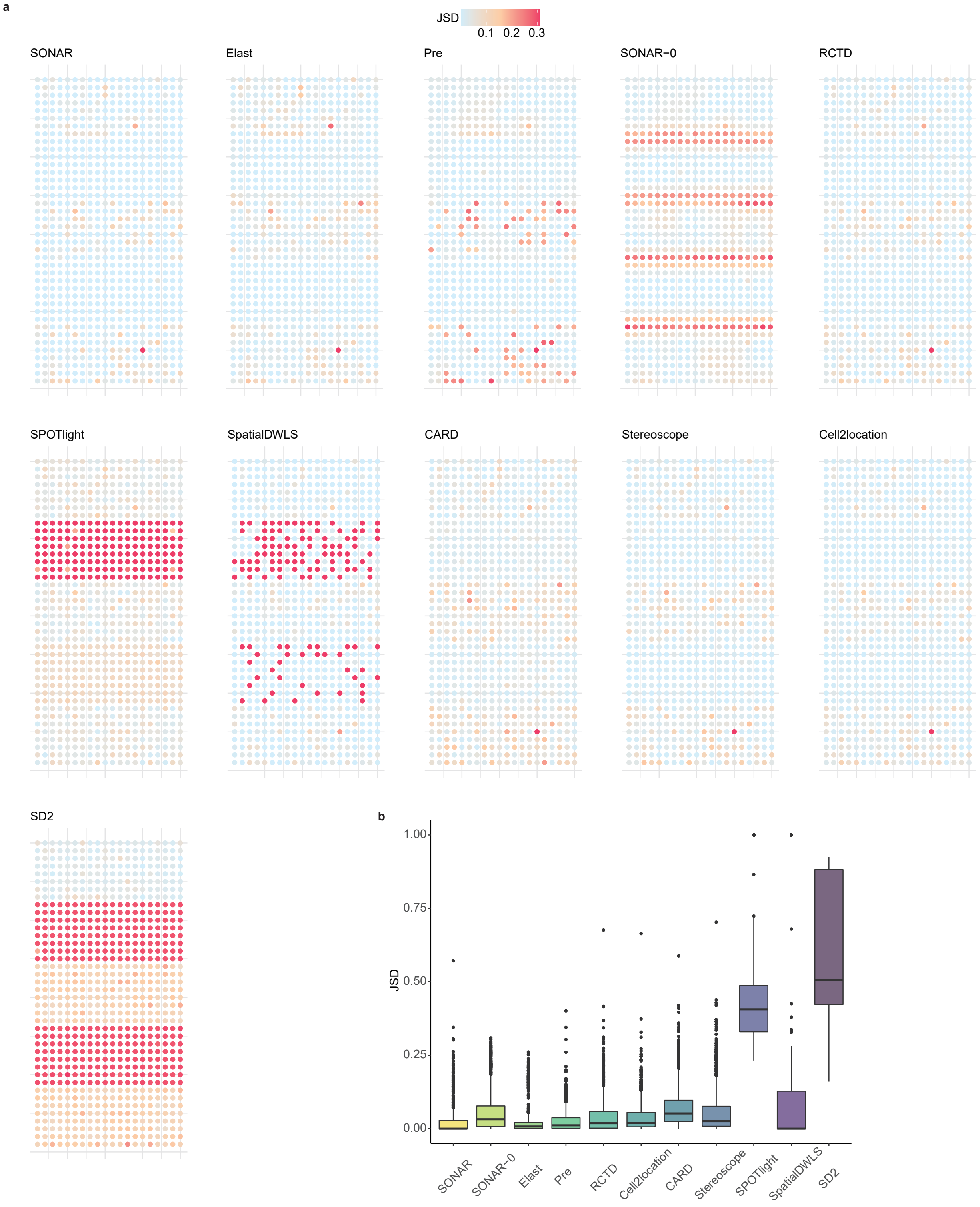

a
JSD
0.1 0.2 0.3
SONAR
Elast
Pre
SONAR−0
RCTD
SPOTlight
SpatialDWLS
CARD
Stereoscope
Cell2location
SD2
b
JSD
1.00
0.75
0.50
0.25
0.00
SONAR
SONAR−0
Elast
Pre
RCTD
Cell2location
CARD
Stereoscope
SPOTlight
SpatialDWLS
SD2

Supplementary Fig. 5 JSD for all spots in layer pattern.
a, we show the JSD spatial distribution of all comparing algorithms for each spot in layer pattern, and the color bar is the JSD value. In **b**, the boxplots show the JSD comparsion for each spot corresponding to **a** (n = 800 spots). Each box plot ranges from the first and third quartiles with the median as the horizontal line, while whiskers represent 1.5 times the interquartile range from the lower and upper bounds of the box. SONAR-0 is the raw version of SONAR that without pre-clustering or elastic weighting. PreC is SONAR-0 only with pre-clustering, Elast is SONAR-0 only with elastic weighting, and SONAR is the complete version. Source data are provided as a Source Data file.

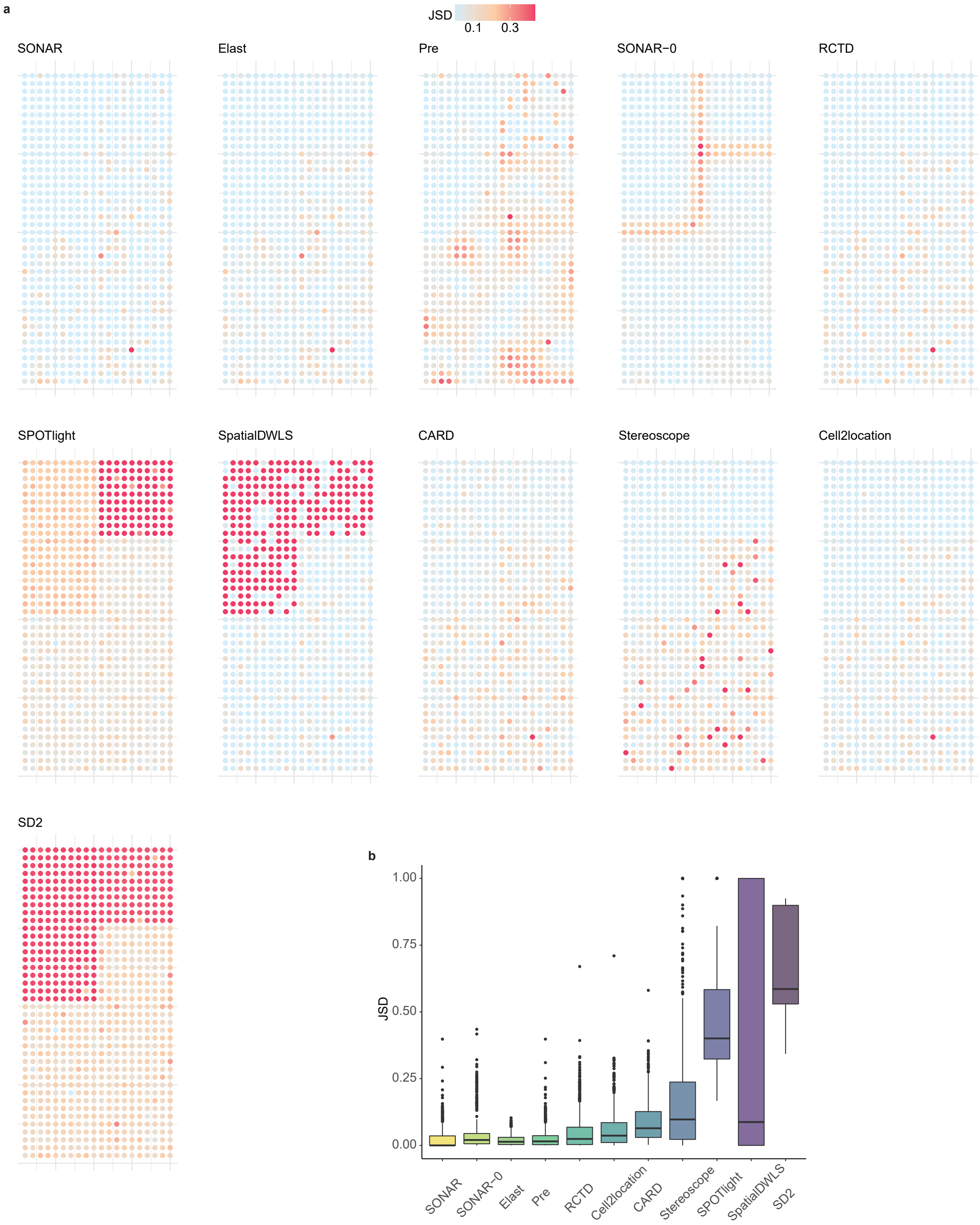
a
JSD
0.1
0.3
SONAR
Elast
Pre
SONAR−0
RCTD
SPOTlight
SpatialDWLS
CARD
Stereoscope
Cell2location
SD2
b
JSD
1.00
0.75
0.50
0.25
0.00
SONAR
SONAR−0
Elast
Pre
RCTD
Cell2location
CARD
Stereoscope
SPOTlight
SpatialDWLS
SD2

Supplementary Fig. 6 JSD for all spots in block pattern.
a, we show the JSD spatial distribution of all comparing algorithms for each spot in block pattern, and the color bar is the JSD value. In **b**, the boxplots show the JSD comparsion for each spot corresponding to **a** (n = 800 spots). Each box plot ranges from the first and third quartiles with the median as the horizontal line, while whiskers represent 1.5 times the interquartile range from the lower and upper bounds of the box. SONAR-0 is the raw version of SONAR that without pre-clustering or elastic weighting. PreC is SONAR-0 only with pre-clustering, Elast is SONAR-0 only with elastic weighting, and SONAR is the complete version. Source data are provided as a Source Data file.

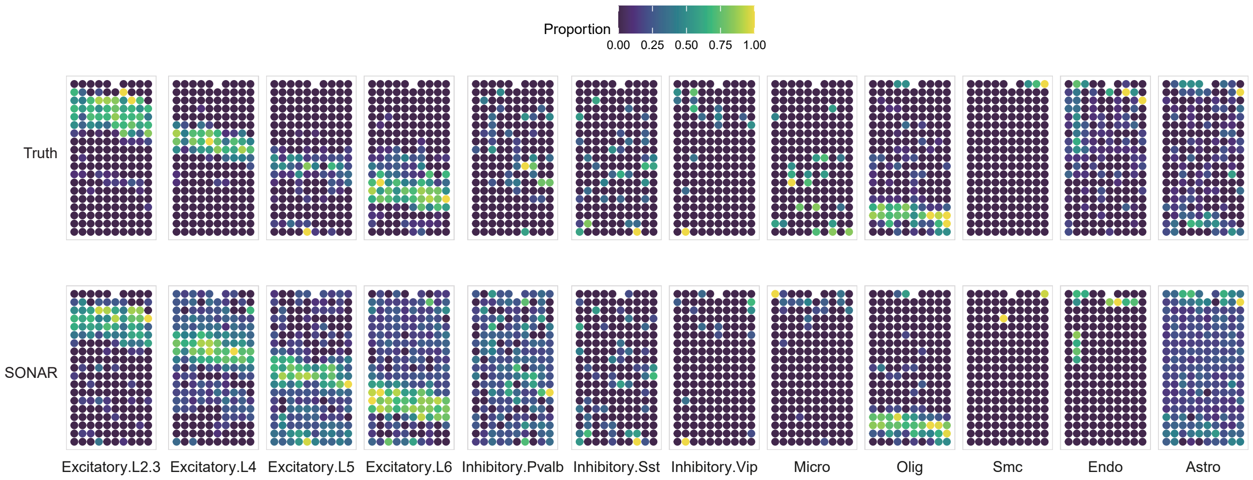

Supplementary Fig. 7 Inferred proportions by SONAR for all cell types in mouse cortex. The ground truth proportions (top) from (4) and corresponding predicted proportions by SONAR (bottom) for 12 cell types. The color is scaled for each type (Min-MaxScaling). Source data are provided as a Source Data file.

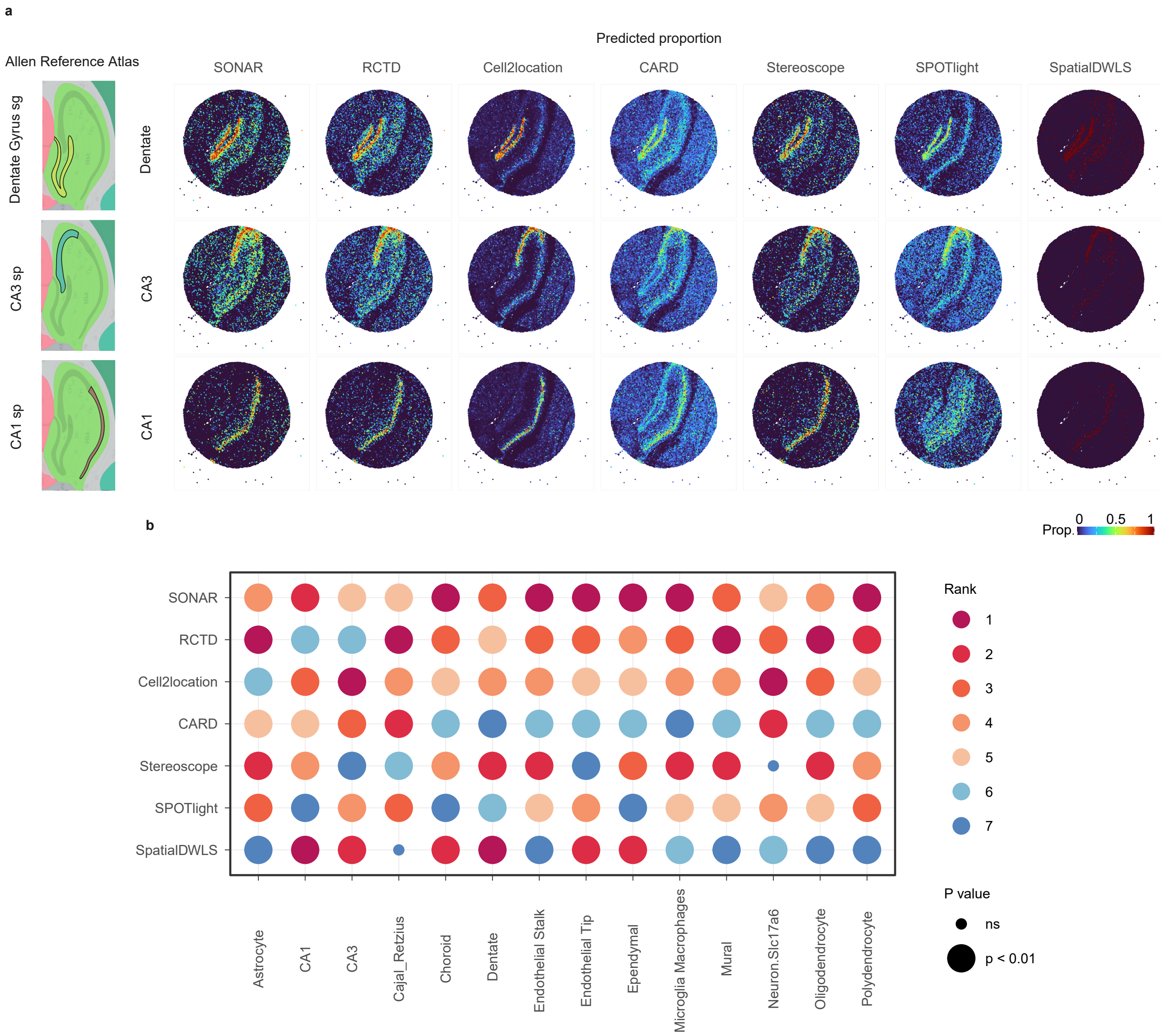

Supplementary Fig. 8 SONAR accurately maps cell types to spatial location on mouse hippocampus Slide-seq V2 dataset.
a, Left: The annotation of hippocampus structures from the Allen Reference Atlas of an adult mouse brain. From bottom to top are CA1 sp, CA3 sp, Dentate Gyrus sg spatial domains in the Allen Reference Atlas. Right: The scaled predicted proportion of dominant cell types on each location inferred from SONAR and all comparing algorithms, including CA1, CA3, and Dentate cell types. **b**, The scatter plot shows the correlations between inferred cell-type proportions and corresponding cell-type-specific marker genes across spatial locations for all comparing algorithms. Rank (color) represents the type-specific descending order of correlations for all algorithms with the p-value (size) tested by a one-sided (greater) Spearman rank correlation test. Source data are provided as a Source Data file including the exact p-values.

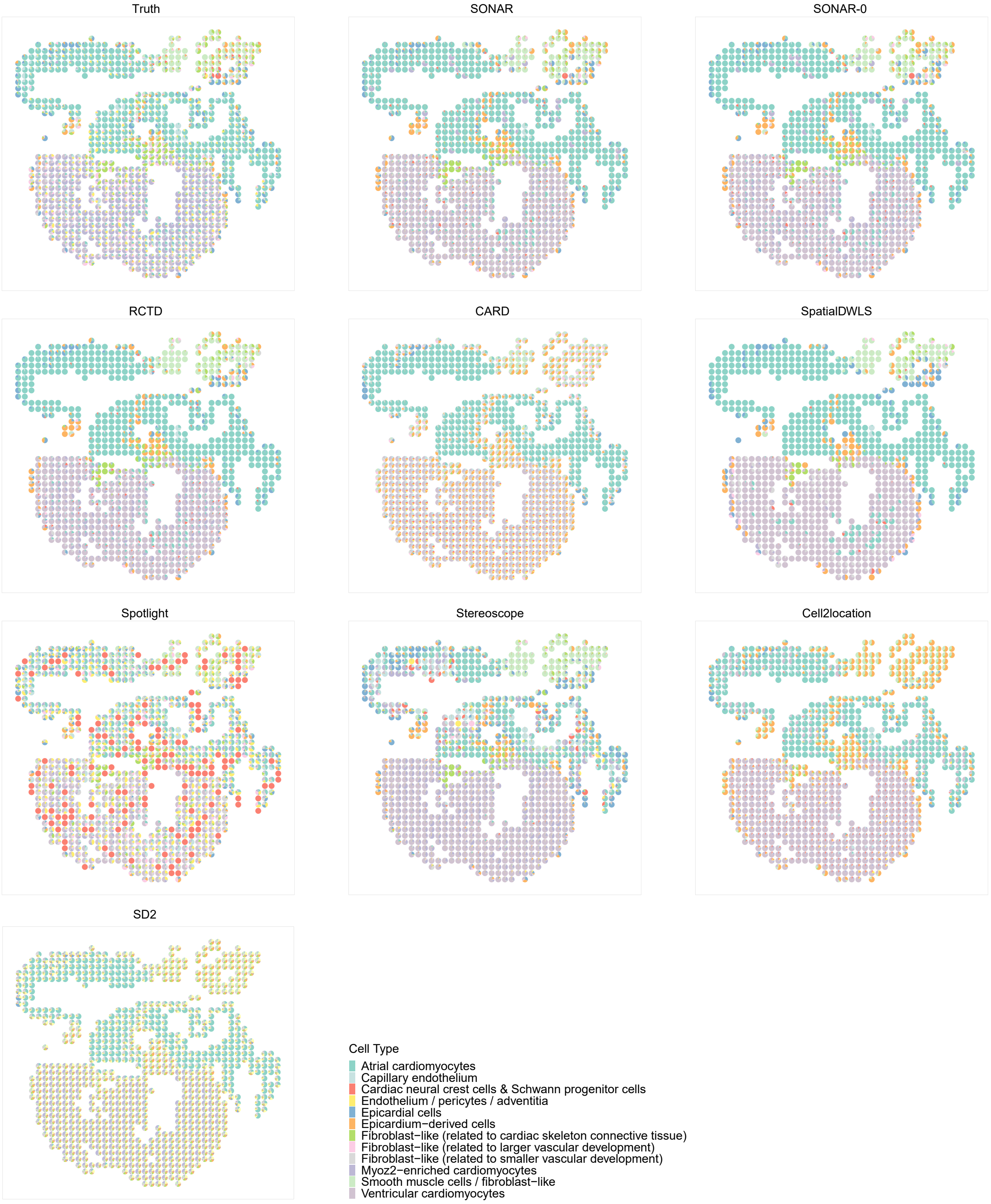
Truth
SONAR
SONAR-0
RCTD
CARD
SpatialDWLS
Spotlight
Stereoscope
Cell2location
SD2
Cell Type
Atrial cardiomyocytes
Capillary endothelium
Cardiac neural crest cells & Schwann progenitor cells
Endothelium / pericytes / adventitia
Epicardial cells
Epicardium−derived cells
Fibroblast−like (related to cardiac skeleton connective tissue)
Fibroblast−like (related to larger vascular development)
Fibroblast−like (related to smaller vascular development)
Myoz2−enriched cardiomyocytes
Smooth muscle cells / fibroblast−like
Ventricular cardiomyocytes

Supplementary Fig. 9 Spatial scatter pie plots for human heart by comparing algorithms.
The spatial scatter pie plots display the ground truth (top 1, left 1) from (5) and the inferred cell-type composition on each spot from different deconvolution methods. Compared deconvolution methods include SONAR, SONAR-0, RCTD(6), CARD(7), SpatialDWLS(8), SPOTlight(9), Stereoscope(10), Cell2location(11), SD2(12). Source data are provided as a Source Data file.

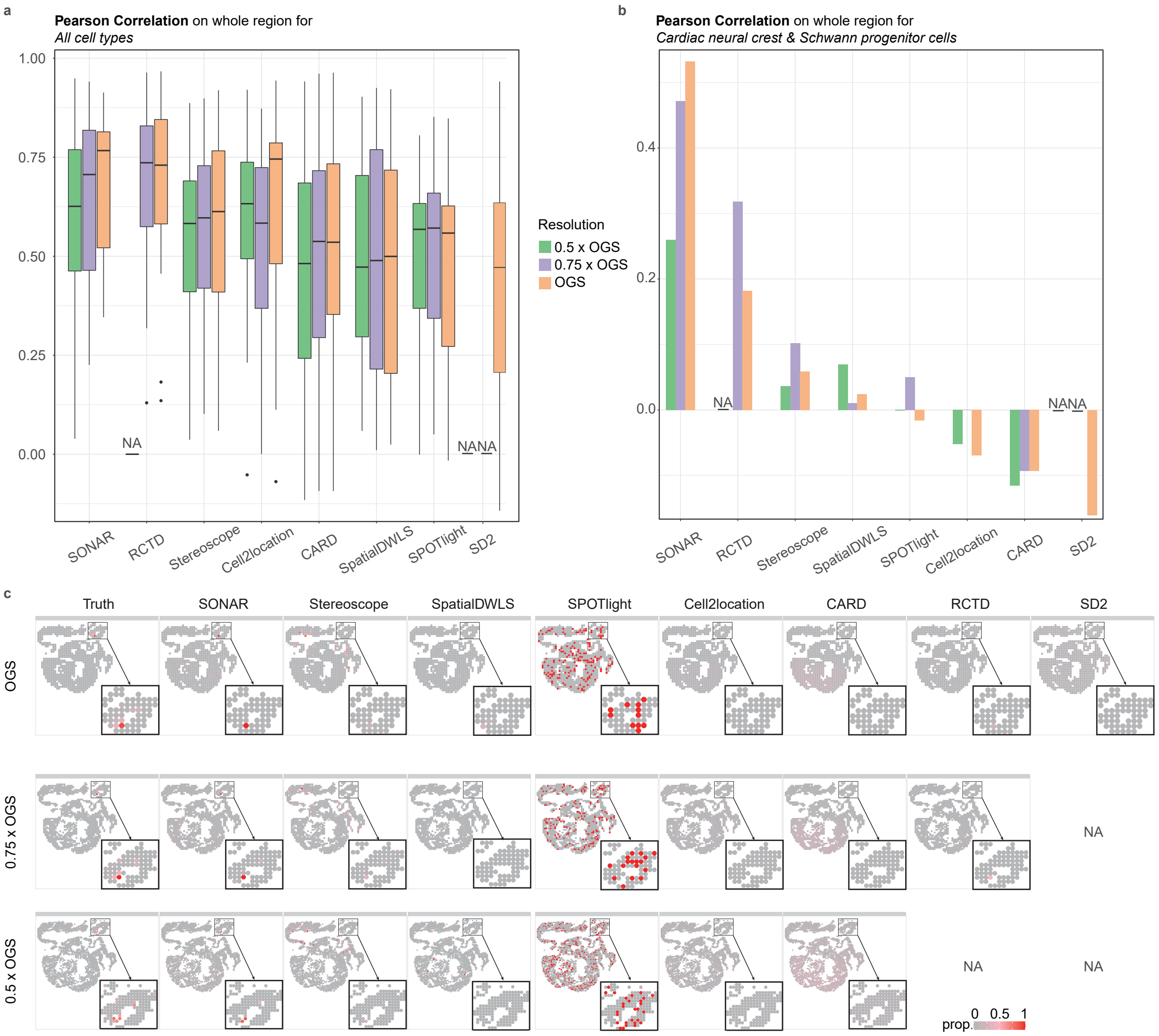

Supplementary Fig. 10 SONAR show robust performance on different spot scale.
a, The boxplot shows the Pearson correlation under the one-sided t test between the predicted proportion and the ground truth on the whole region for all cell types under three different resolutions (n = 12 cell types). The color denotes datasets with different resolutions. Each box plot ranges from the first and third quartiles with the median as the horizontal line, while whiskers represent 1.5 times the interquartile range from the lower and upper bounds of the box. **b**, The barplot shows the Pearson correlation under the one-sided t test between the predicted proportion and the ground truth on the whole region for Cardiac neural crest and Schwann progenitor cells under three different resolutions. **c**, The ground truth and scaled predicted proportions of Cardiac neural crest and Schwann progenitor cells inferred by comparing algorithms are displayed on each spot for three datasets with different resolution. Selected area is the magnified image of outflow tract (OFT). OGS is an abbreviation for original grid size. SD2 did not converge during the training of the graph model with datasets with reduced grid size. RCTD failed to return result in dataset with 50% of original grid size. Source data are provided as a Source Data file.

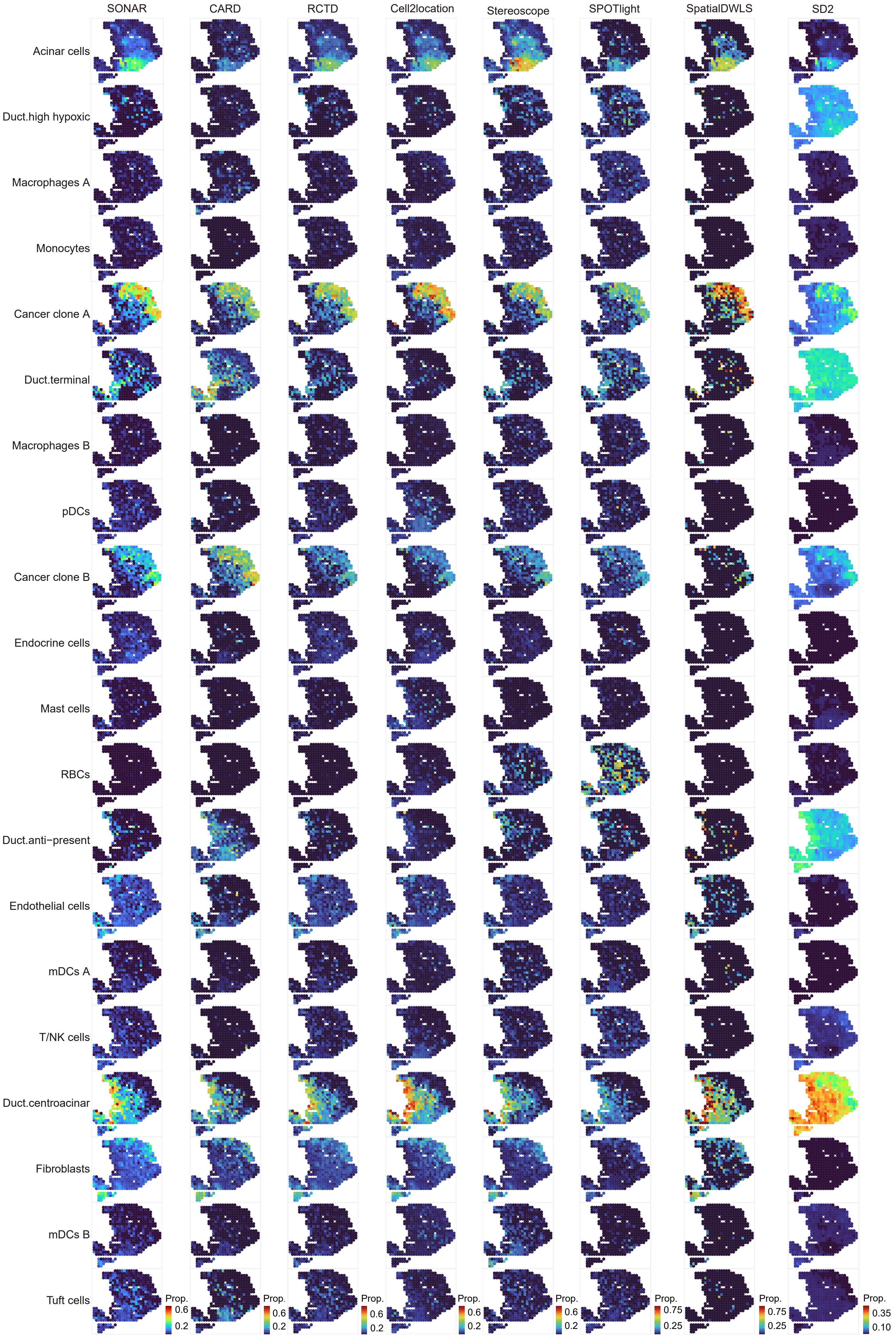

SONAR
CARD
RCTD
Cell2location
Stereoscope
SPOTlight
SpatialDWLS
SD2
Acinar cells
Duct.high hypoxic
Macrophages A
Monocytes
Cancer clone A
Duct.terminal
Macrophages B
pDCs
Cancer clone B
Endocrine cells
Mast cells
RBCs
Duct.anti−present
Endothelial cells
mDCs A
T/NK cells
Duct.centroacinar
Fibroblasts
mDCs B
Tuft cells
Prop.
0.6
0.2
0.75
0.25
0.35
0.10

Supplementary Fig. 11 Inferred cell types proportions for PDAC by comparing algorithms.

The proportions of each of the cell types inferred by other comparing algorithms are displayed on each spot. mDCs, myeloid dendritic cells; pDCs, plasmacytoid dendritic cells; RBCs, red blood cells; NK cells, natural killer cells; Duct.high hypoxic, Ductal high hypoxic; Duct.anti-present, Ductal antigen-presenting; Duct.centroacinar, Ductal centroacinar; Duct.terminal, Ductal terminal. Source data are provided as a Source Data file.

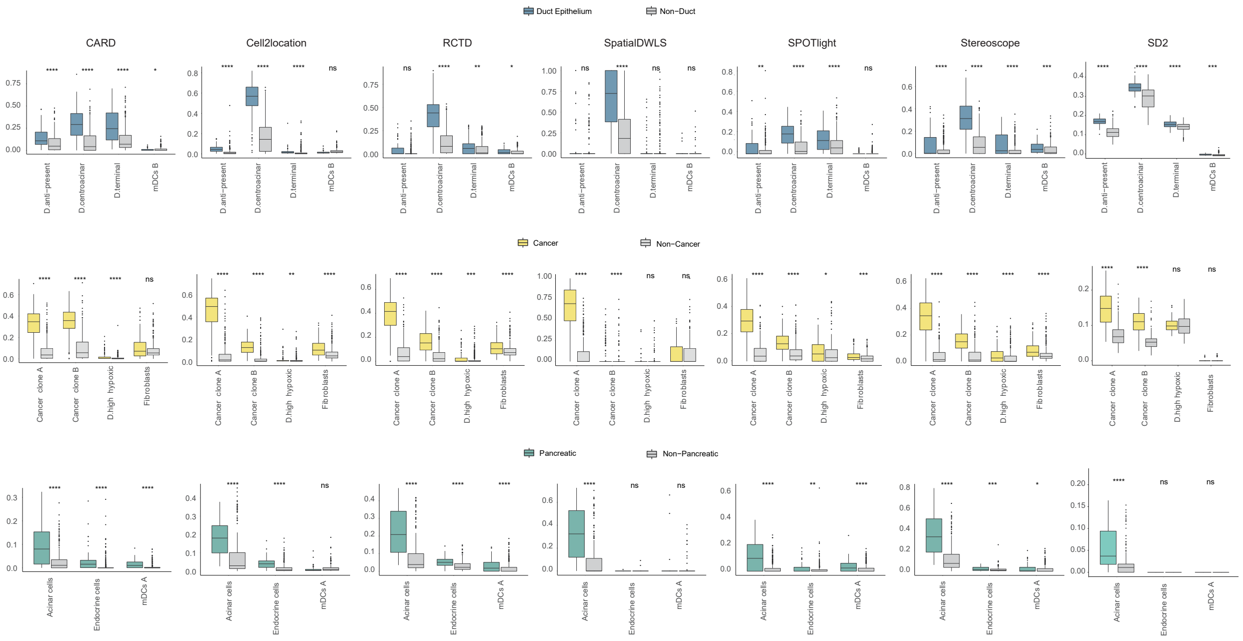

Supplementary Fig. 12 Comparisons of regional major types prediction by comparing algorithms.
Comparisons of regional major types proportions inferred by all comparing algorithms in three regions: Duct Epithelium region (n = 72 spots) versus non-Duct Epithelium region (n = 354 spots) (top), Cancer region (n = 137 spots) versus non-Cancer region (n = 289 spots) (middle), Pancreatic region (n = 70 spots) versus non-Pancreatic region (n = 356 spots) (bottom), with the p-value tested by a one-sided (greater) Wilcoxon rank sum test. ****: $p \leq 0.0001$, ***: $p \leq 0.001$, **: $p \leq 0.01$, *: $p \leq 0.05$. mDCs, myeloid dendritic cells; D.high hypoxic, Ductal high hypoxic; D.anti-present, Ductal antigen-presenting; D.centroacinar, Ductal centroacinar; D.terminal, Ductal terminal; Non-Duct, Non-Duct Epithelium. Each box plot ranges from the first and third quartiles with the median as the horizontal line, while whiskers represent 1.5 times the interquartile range from the lower and upper bounds of the box. Source data are provided as a Source Data file.

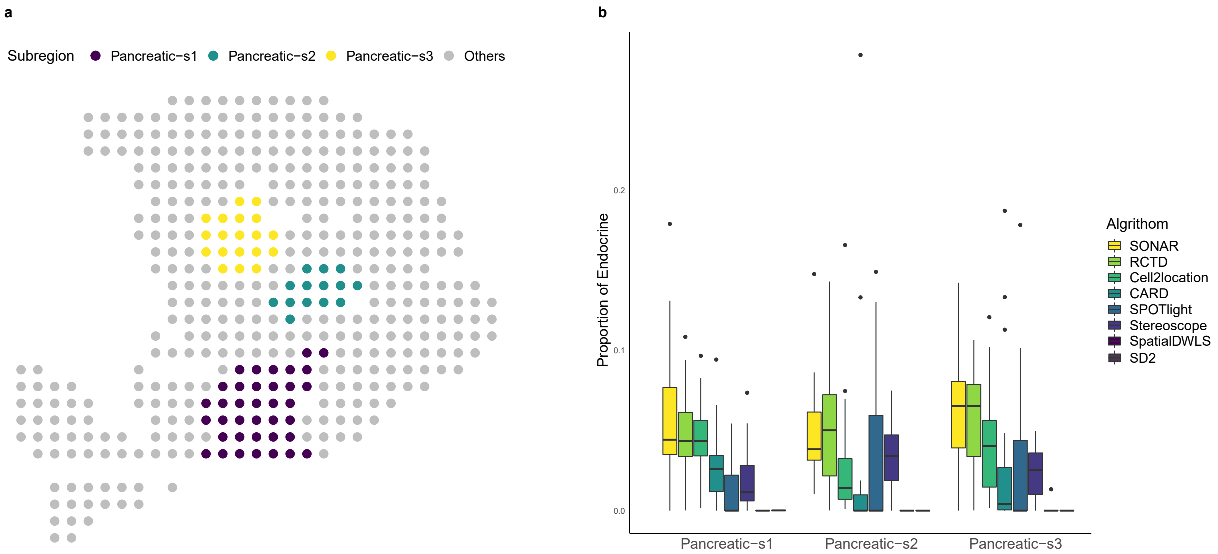

Supplementary Fig. 13 The predicted local proportion of Endocrine cells in pancreatic subregions.

a, The annotation of pancreatic subregions, including pancreatic-s1 (bottom part of pancreatic region), pancreatic-s2 (middle part of pancreatic region), pancreatic-s3 (top part of pancreatic region). **b**, The predicted local proportion of Endocrine cells for each spot in pancreatic subregions by comparing algorithms (n = 37, 14, 19 spots for three subregions respectively). Each box plot ranges from the first and third quartiles with the median as the horizontal line, while whiskers represent 1.5 times the interquartile range from the lower and upper bounds of the box. Source data are provided as a Source Data file.

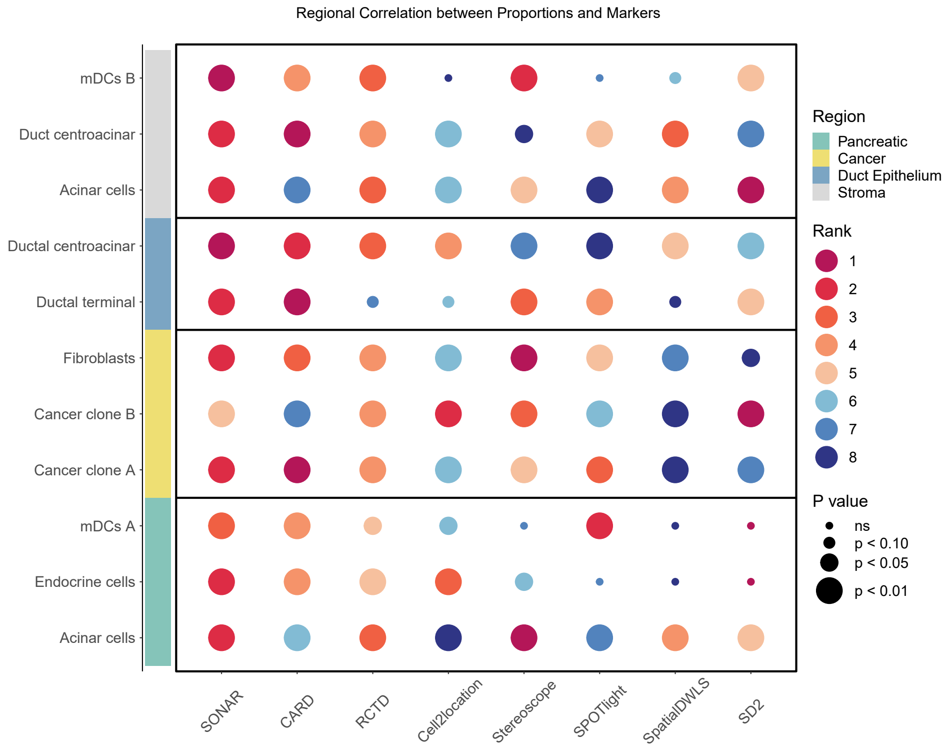

Supplementary Fig. 14 Regional correlations between inferred cell-type proportions by SONAR and corresponding marker genes.
The scatter plot shows the correlations between inferred cell-type proportions and corresponding cell-type-specific marker genes across spatial locations in 4 regions (Pancreatic, Cancer, Duct Epithelium, Stroma) for SONAR. Rank (color) represents the type-specific descending order of correlation for all algorithms with the p-value (size) tested by a one-sided (greater) Spearman rank test. Source data are provided as a Source Data file including the exact p-values.

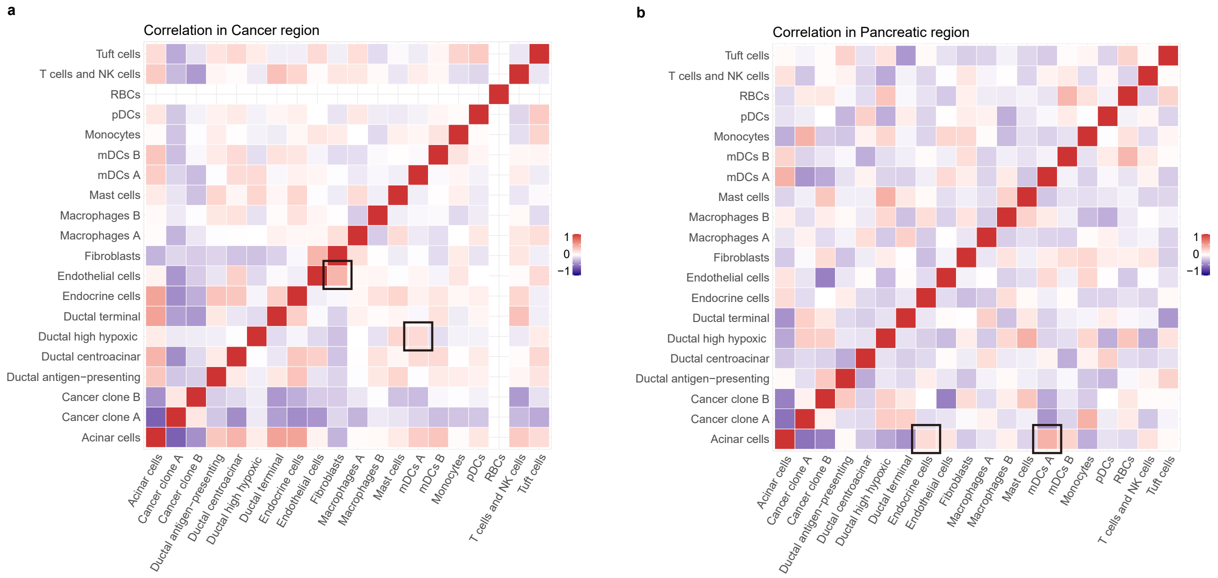

Supplementary Fig. 15 Co-localization in the Cancer region and Pancreatic region. **a**, Correlations in cell-type proportion across spatial locations in Cancer region between pairs of cell types inferred by SONAR. **b**, Correlations in cell-type proportion across spatial locations in Pancreatic region between pairs of cell types inferred by SONAR. The color is scaled by the Pearson correlation value under two-sided t test. The black boxes show four pairs cell types co-localization. Source data are provided as a Source Data file.

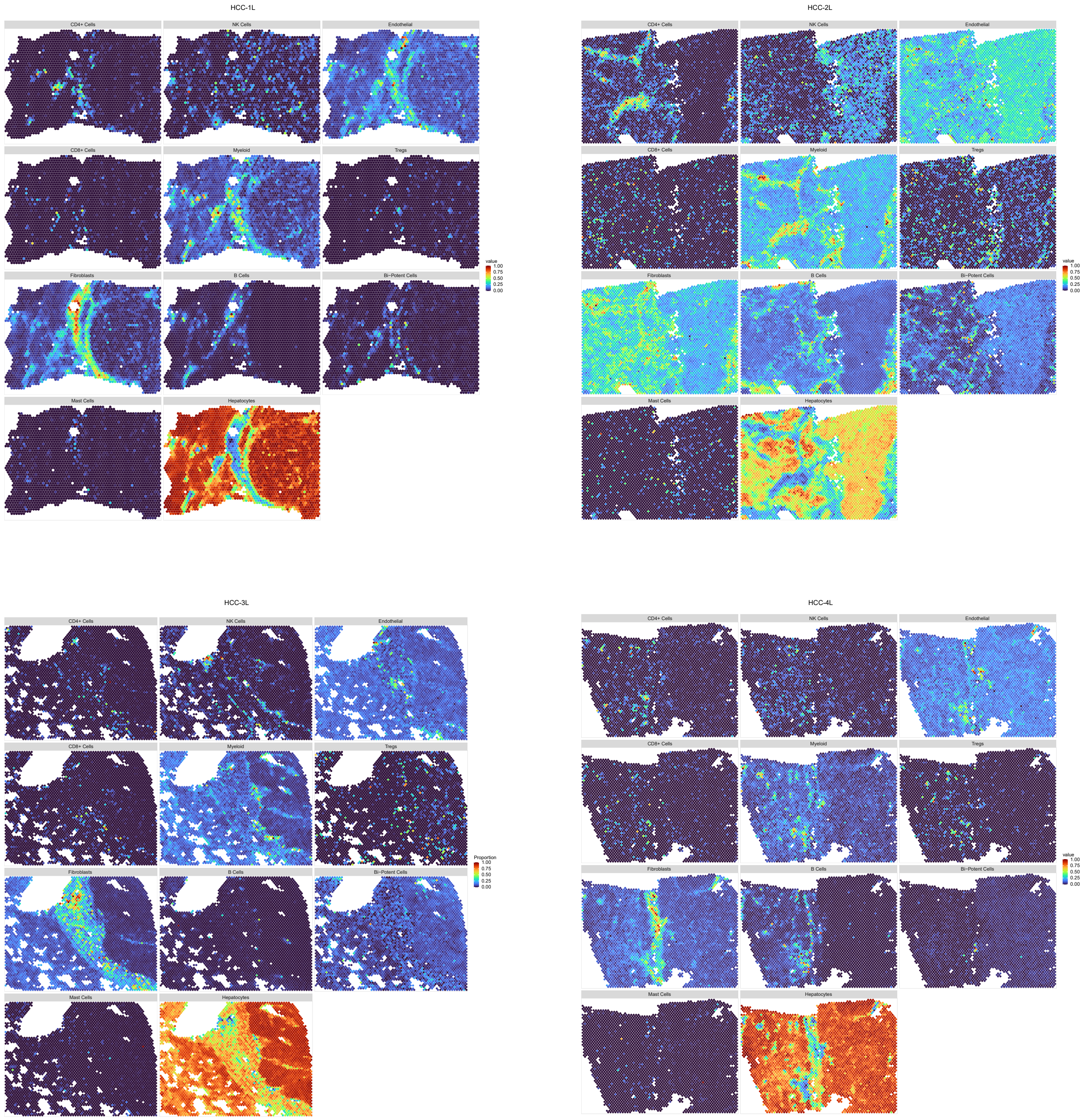
HCC-1L
CD4+ Cells
NK Cells
Endothelial
CD8+ Cells
Myeloid
Tregs
Fibroblasts
B Cells
Bi−Potent Cells
Mast Cells
Hepatocytes
value
1.00
0.75
0.50
0.25
0.00
HCC-2L
CD4+ Cells
NK Cells
Endothelial
CD8+ Cells
Myeloid
Tregs
Fibroblasts
B Cells
Bi−Potent Cells
Mast Cells
Hepatocytes
value
1.00
0.75
0.50
0.25
0.00
HCC-3L
CD4+ Cells
NK Cells
Endothelial
CD8+ Cells
Myeloid
Tregs
Fibroblasts
B Cells
Bi−Potent Cells
Mast Cells
Hepatocytes
Proportion
1.00
0.75
0.50
0.25
0.00
HCC-4L
CD4+ Cells
NK Cells
Endothelial
CD8+ Cells
Myeloid
Tregs
Fibroblasts
B Cells
Bi−Potent Cells
Mast Cells
Hepatocytes
value
1.00
0.75
0.50
0.25
0.00

Supplementary Fig. 16 The spatial scatter plots of proportions for all cell types in 4 patients inferred by SONAR.
The spatial scatter plots show the proportions of all cell types in 4 patients for each spatial location inferred by SONAR. The colors are scaled for each type (Min-MaxScaling). Source data are provided as a Source Data file.

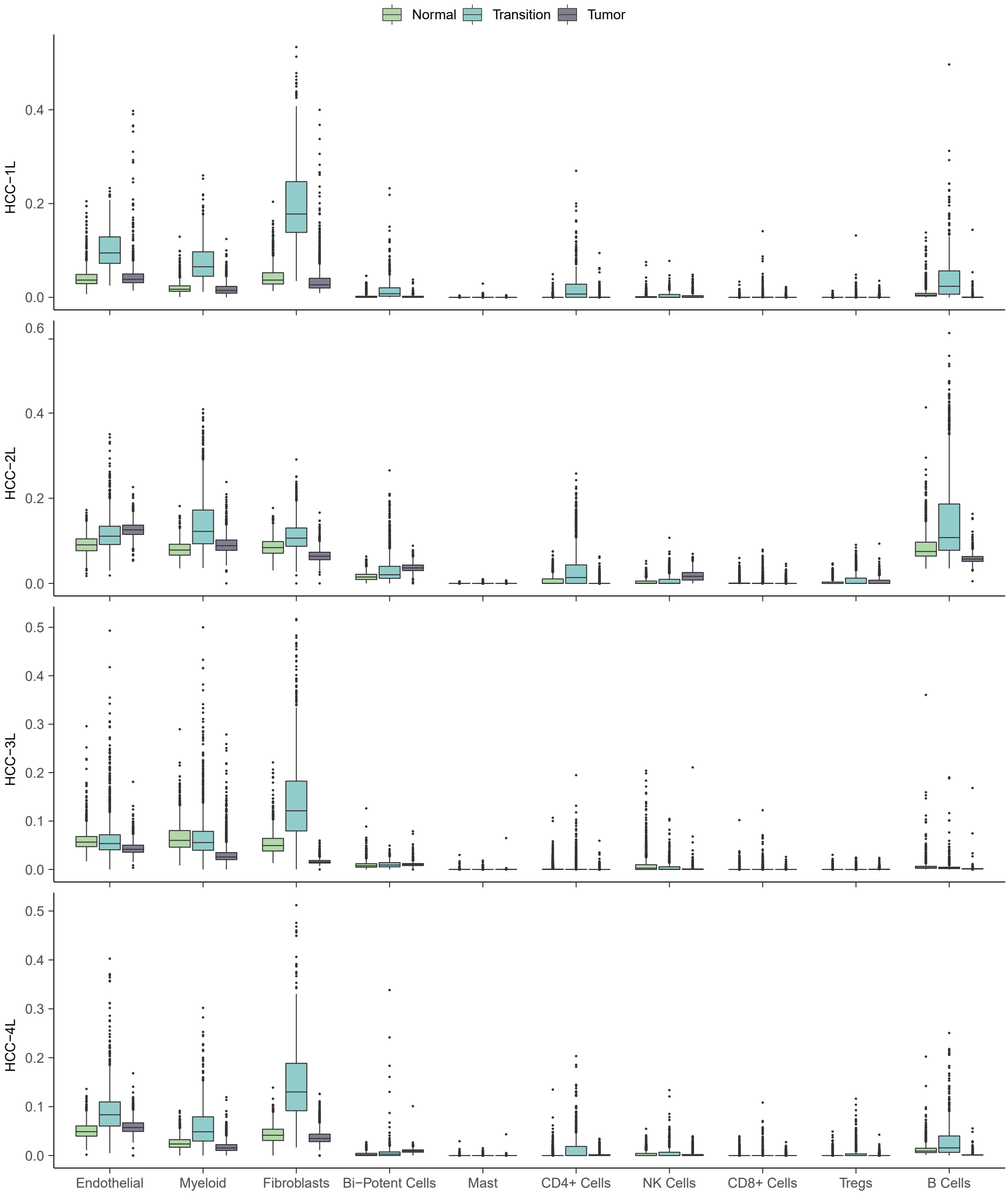

Supplementary Fig. 17 The regional predicted proportions by SONAR of all cell types for 4 patients.

The regional proportions of all cell types inferred by SONAR for 4 patients are presented (n = 1,064; 1,063; 1,684; 1,381; 409; 1,922; 1,083; 601; 1,281; 1,685; 1,311; 2,127 spots for Normal of HCC-1L to HCC-4L, Transition of HCC-1L to HCC-4L and Tumor of HCC-1L to HCC-4L, respectively). Each box plot ranges from the first and third quartiles with the median as the horizontal line, while whiskers represent 1.5 times the interquartile range from the lower and upper bounds of the box. Source data are provided as a Source Data file.

Prediction of Fibroblasts and Endothelial for 4 patients

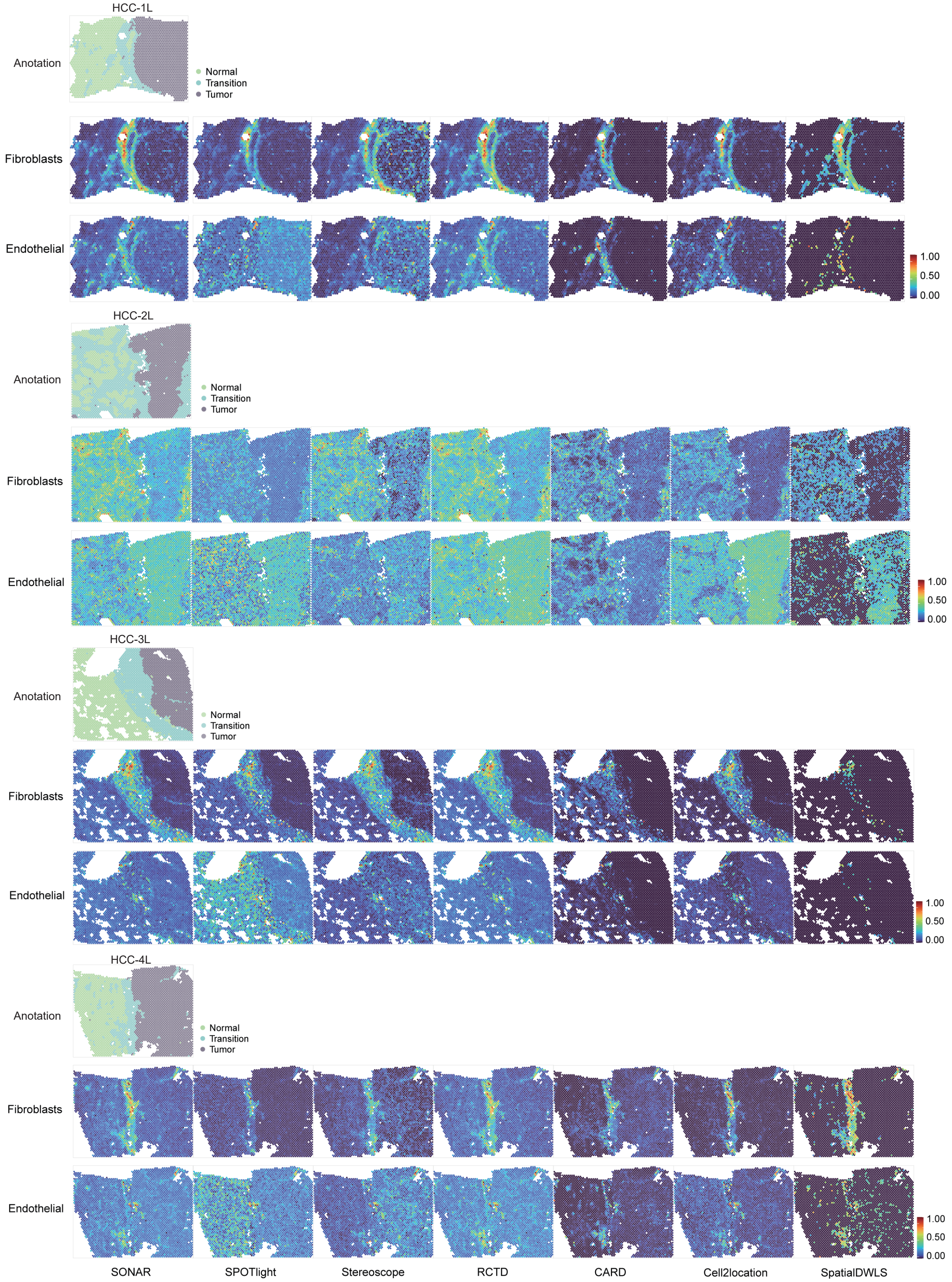

Supplementary Fig. 18 Predicted proportions of Fibroblasts and Endothelial for all algorithms in 4 patients.

In each group: the top row represents region annotation formed by Normal, Transition, and Tumor regions. The following rows represent the (scaled) proportions of Fibroblasts and Endothelial cells inferred by all comparing algorithms. From first group to forth group represents different patients: HCC-1L, HCC-2L, HCC-3L, HCC-4L. Source data are provided as a Source Data file.

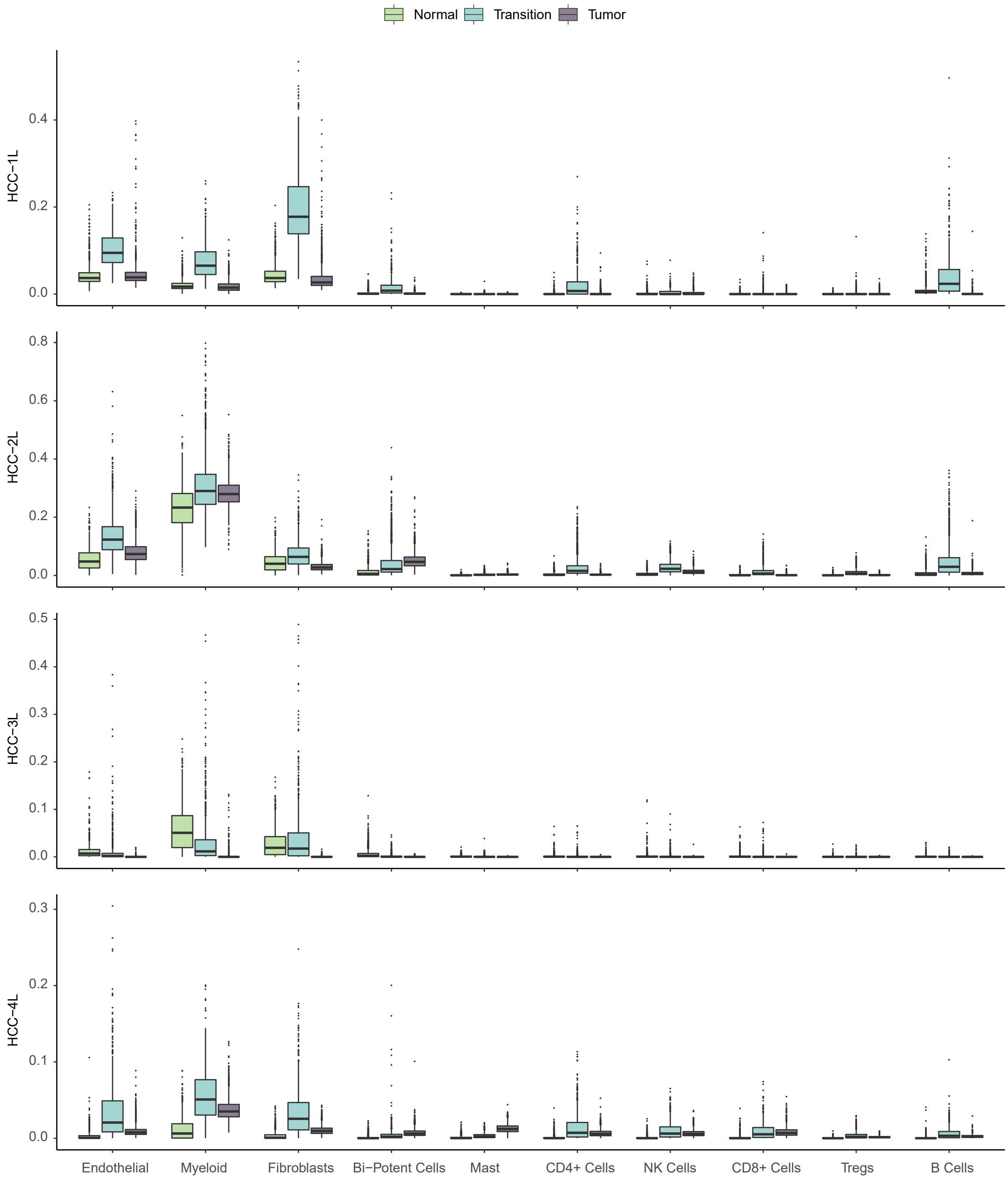

Supplementary Fig. 19 The regional predicted proportions by CARD of all cell types for 4 patients.

The regional proportions of all cell types inferred by CARD for 4 patients are presented (n = 1,064; 1,063; 1,684; 1,381; 409; 1,922; 1,083; 601; 1,281; 1,685; 1,311; 2,127 spots for Normal of HCC-1L to HCC-4L, Transition of HCC-1L to HCC-4L and Tumor of HCC-1L to HCC-4L, respectively). Each box plot ranges from the first and third quartiles with the median as the horizontal line, while whiskers represent 1.5 times the interquartile range from the lower and upper bounds of the box. Source data are provided as a Source Data file.

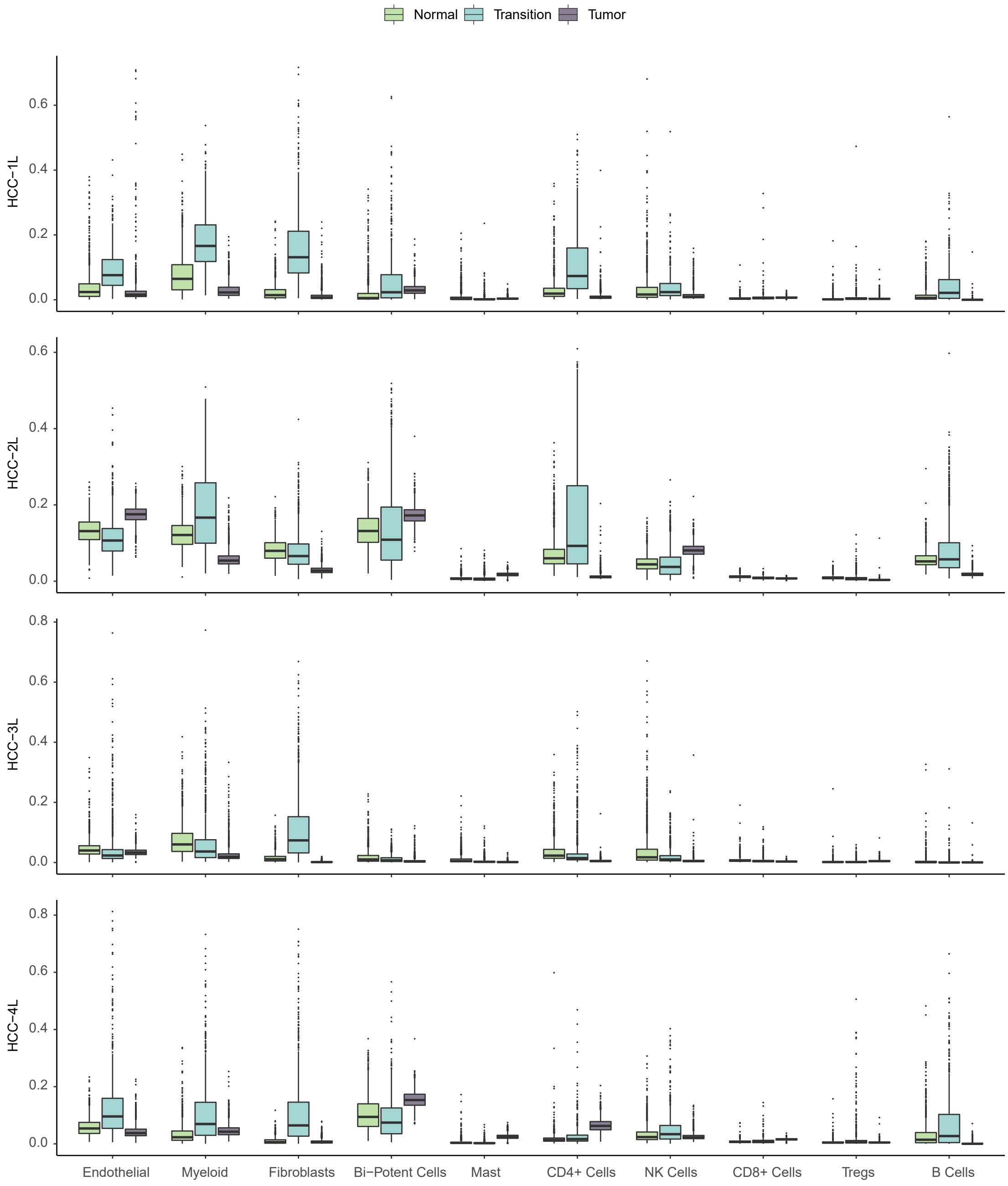

Supplementary Fig. 20 The regional predicted proportions by Cell2location of all cell types for 4 patients.
The regional proportions of all cell types inferred by Cell2location for 4 patients are presented (n = 1,064; 1,063; 1,684; 1,381; 409; 1,922; 1,083; 601; 1,281; 1,685; 1,311; 2,127 spots for Normal of HCC-1L to HCC-4L, Transition of HCC-1L to HCC-4L and Tumor of HCC-1L to HCC-4L, respectively). Each box plot ranges from the first and third quartiles with the median as the horizontal line, while whiskers represent 1.5 times the interquartile range from the lower and upper bounds of the box. Source data are provided as a Source Data file.

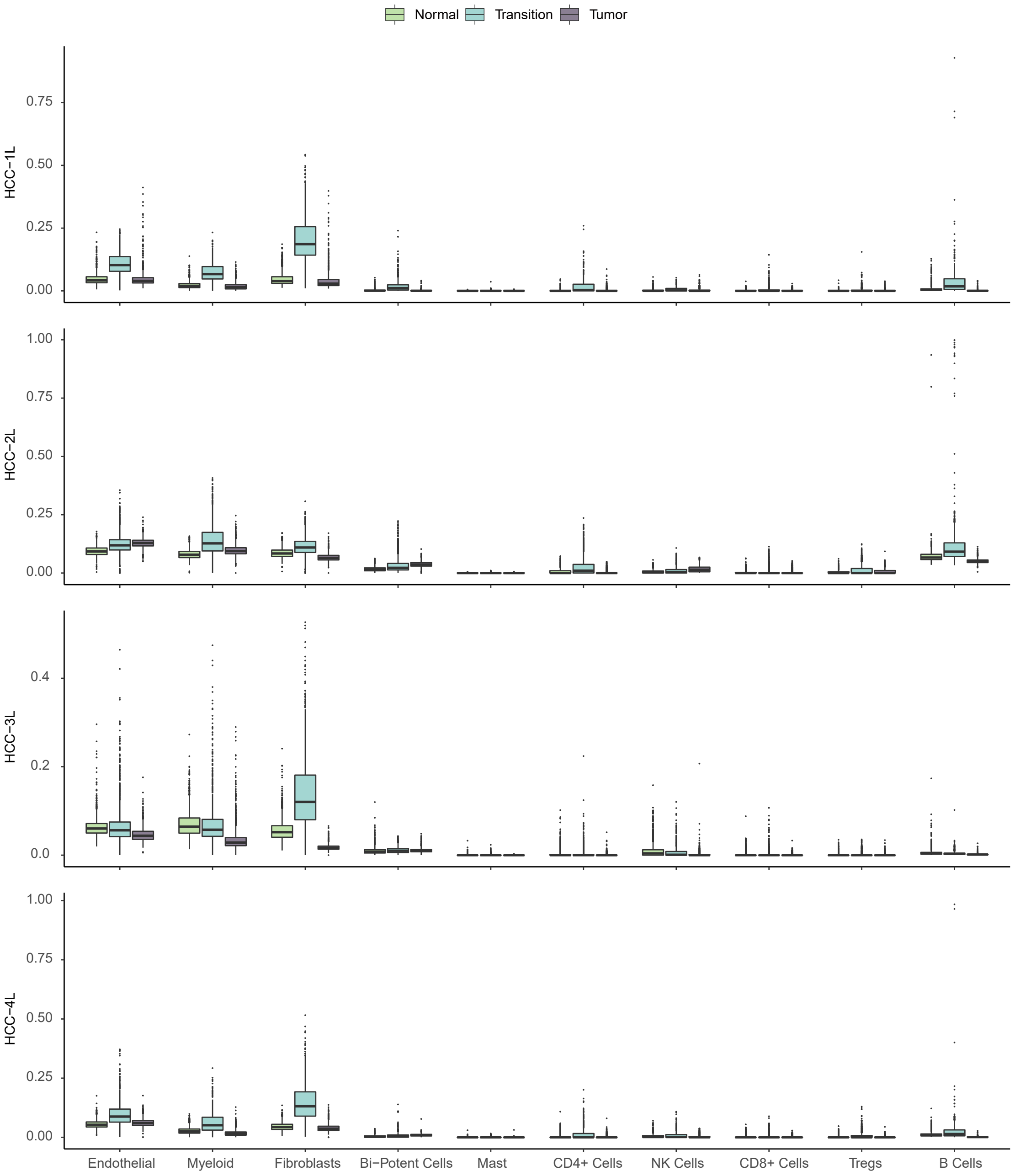

Supplementary Fig. 21 The regional predicted proportions by RCTD of all cell types for 4 patients.

The regional proportions of all cell types inferred by RCTD for 4 patients are presented (n = 1,064; 1,063; 1,684; 1,381; 409; 1,922; 1,083; 601; 1,281; 1,685; 1,311; 2,127 spots for Normal of HCC-1L to HCC-4L, Transition of HCC-1L to HCC-4L and Tumor of HCC-1L to HCC-4L, respectively). Each box plot ranges from the first and third quartiles with the median as the horizontal line, while whiskers represent 1.5 times the interquartile range from the lower and upper bounds of the box. Source data are provided as a Source Data file.

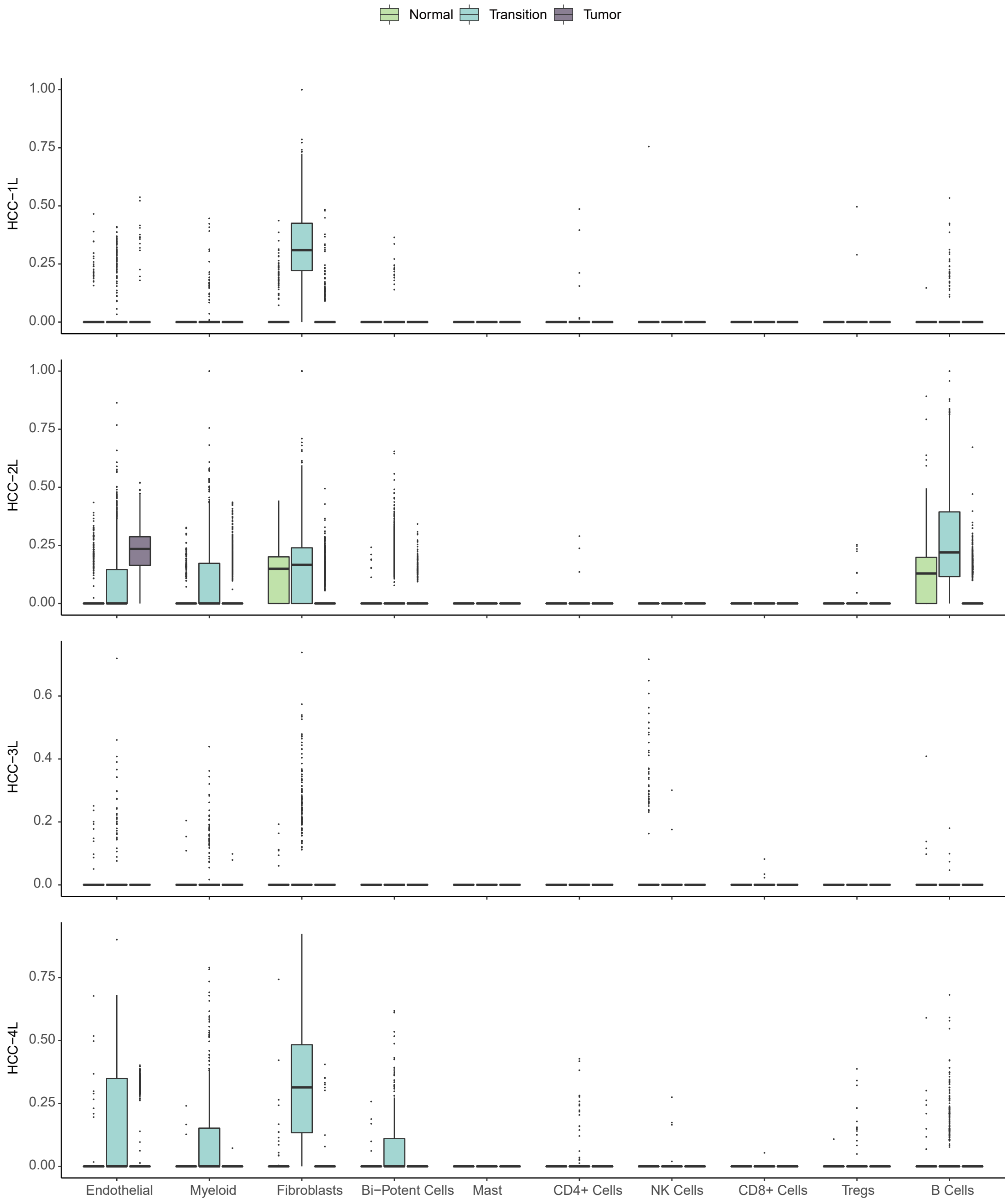

Supplementary Fig. 22 The regional predicted proportions by SpatialDWLS of all cell types for 4 patients.

The regional proportions of all cell types inferred by SpatialDWLS for 4 patients are presented (n = 1,064; 1,063; 1,684; 1,381; 409; 1,922; 1,083; 601; 1,281; 1,685; 1,311; 2,127 spots for Normal of HCC-1L to HCC-4L, Transition of HCC-1L to HCC-4L and Tumor of HCC-1L to HCC-4L, respectively). Each box plot ranges from the first and third quartiles with the median as the horizontal line, while whiskers represent 1.5 times the interquartile range from the lower and upper bounds of the box. Source data are provided as a Source Data file.

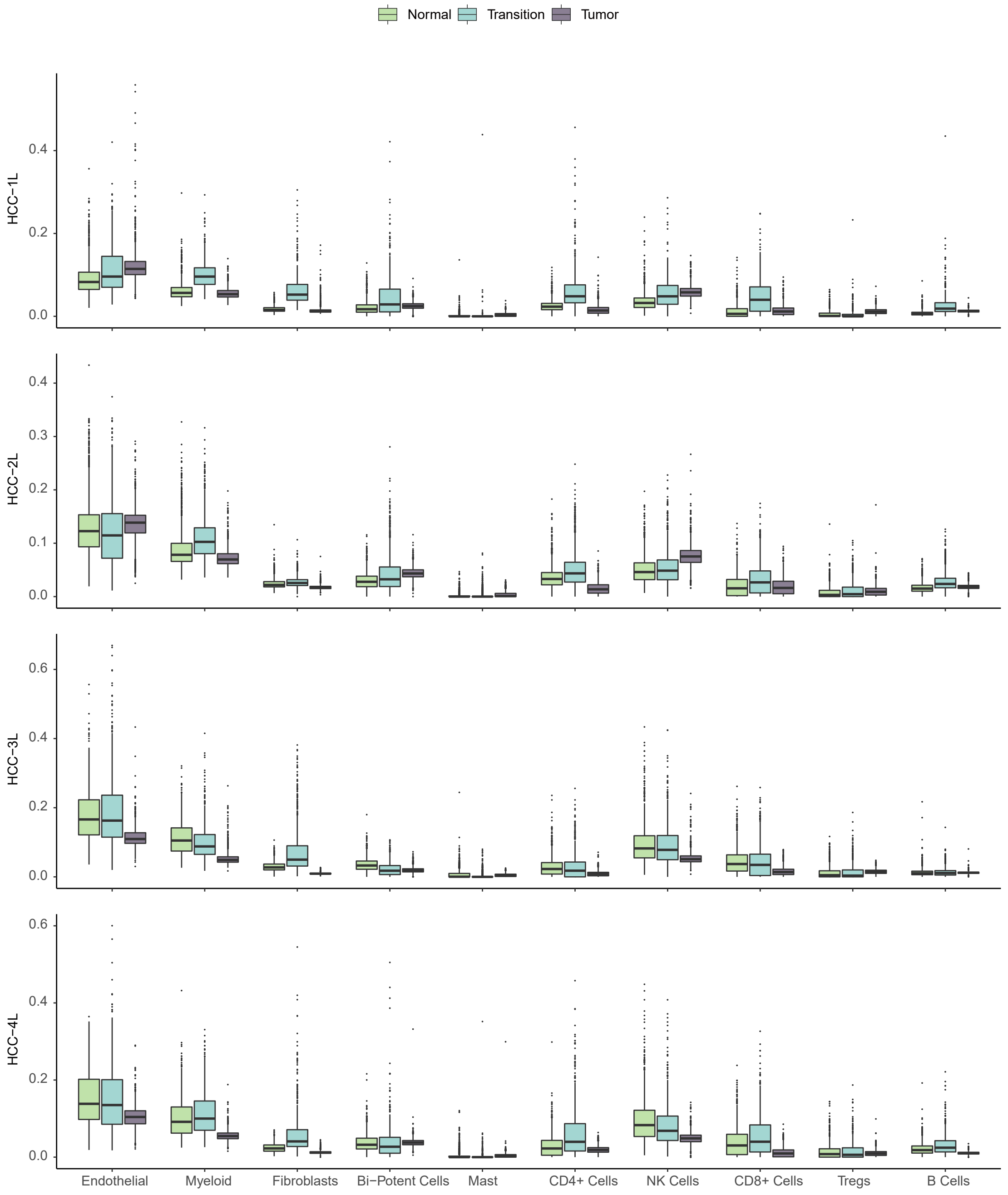

Supplementary Fig. 23 The regional predicted proportions by SPOTlight of all cell types for 4 patients.

The regional proportions of all cell types inferred by SPOTlight for 4 patients are presented (n = 1,064; 1,063; 1,684; 1,381; 409; 1,922; 1,083; 601; 1,281; 1,685; 1,311; 2,127 spots for Normal of HCC-1L to HCC-4L, Transition of HCC-1L to HCC-4L and Tumor of HCC-1L to HCC-4L, respectively). Each box plot ranges from the first and third quartiles with the median as the horizontal line, while whiskers represent 1.5 times the interquartile range from the lower and upper bounds of the box. Source data are provided as a Source Data file.

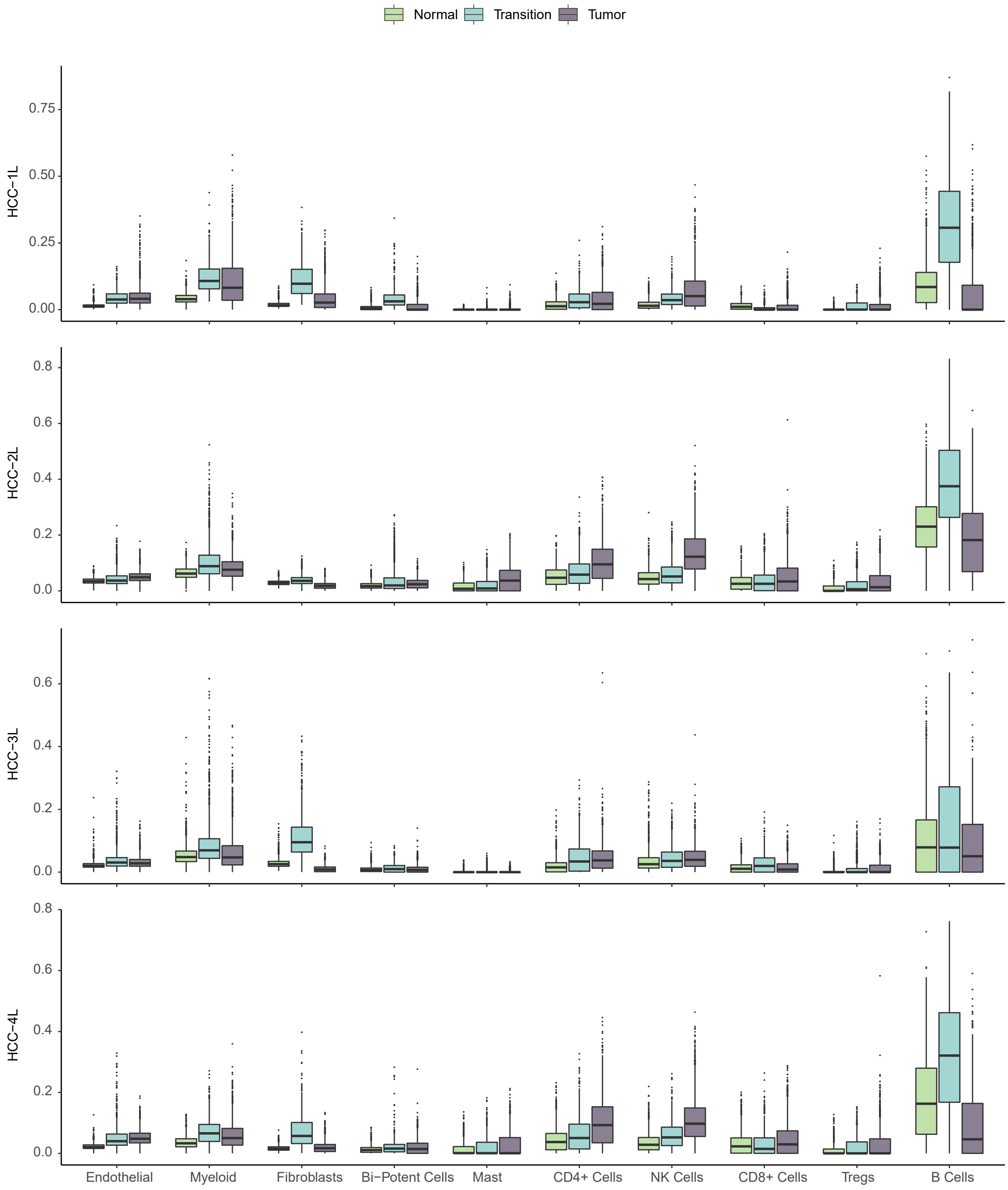

Supplementary Fig. 24 The regional predicted proportions by Stereoscope of all cell types for 4 patients.

The regional proportions of all cell types inferred by Stereoscope for 4 patients are presented (n = 1,064; 1,063; 1,684; 1,381; 409; 1,922; 1,083; 601; 1,281; 1,685; 1,311; 2,127 spots for Normal of HCC-1L to HCC-4L, Transition of HCC-1L to HCC-4L and Tumor of HCC-1L to HCC-4L, respectively). Each box plot ranges from the first and third quartiles with the median as the horizontal line, while whiskers represent 1.5 times the interquartile range from the lower and upper bounds of the box. Source data are provided as a Source Data file.

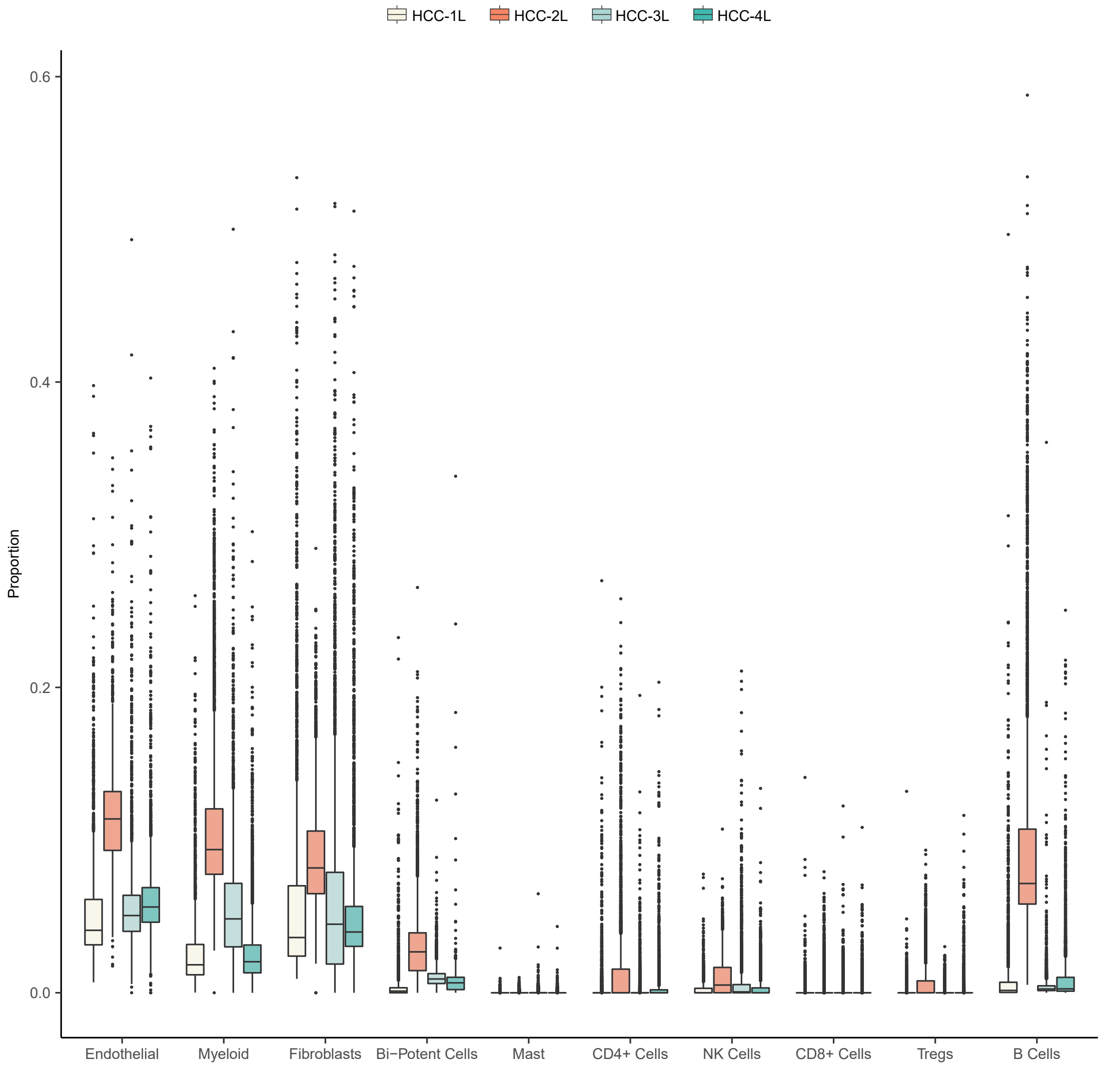

Supplementary Fig. 25 The total predicted proportions of all cell types for 4 patients. The total proportions of all cell types inferred by SONAR for 4 patients are presented (n= 2,754; 4,670; 4,078; 4,109 spots for HCC-1L to HCC-4L respectively). Each box plot ranges from the first and third quartiles with the median as the horizontal line, while whiskers represent 1.5 times the interquartile range from the lower and upper bounds of the box. Source data are provided as a Source Data file.

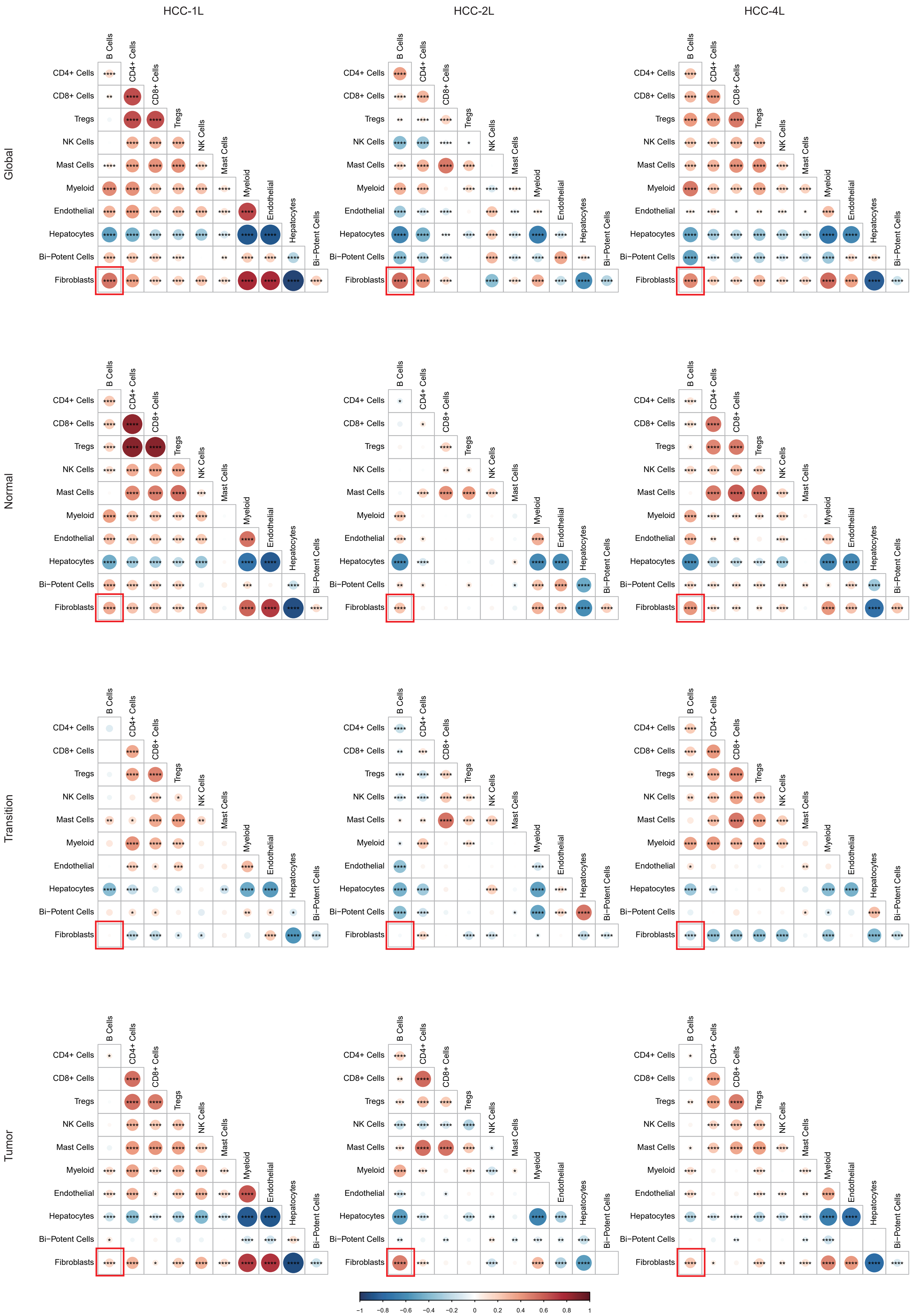

HCC-1L
HCC-2L
HCC-4L
Global
Normal
Transition
Tumor
B Cells
CD4+ Cells
CD8+ Cells
Tregs
NK Cells
Mast Cells
Myeloid
Endothelial
Hepatocytes
Bi−Potent Cells
Fibroblasts
−1
−0.8
−0.6
−0.4
−0.2
0
0.2
0.4
0.6
0.8
1

Supplementary Fig. 26 Global and regional co-localization in 3 patients.
Spearman rank correlations in cell-type proportion across spatial locations in global (first row) and 3 local regions (other rows) between pairs of cell types inferred by SONAR, with the p-value tested by a one-sided (greater) test. ****: $p \leq 0.0001$, ***: $p \leq 0.001$, **: $p \leq 0.01$, *: $p \leq 0.05$. The color represents the correlation values, and the size displays the absolute correlation values. The red boxes indicate the co-localization between the Fibroblasts and B Cells. Source data are provided as a Source Data file including the exact p-values.

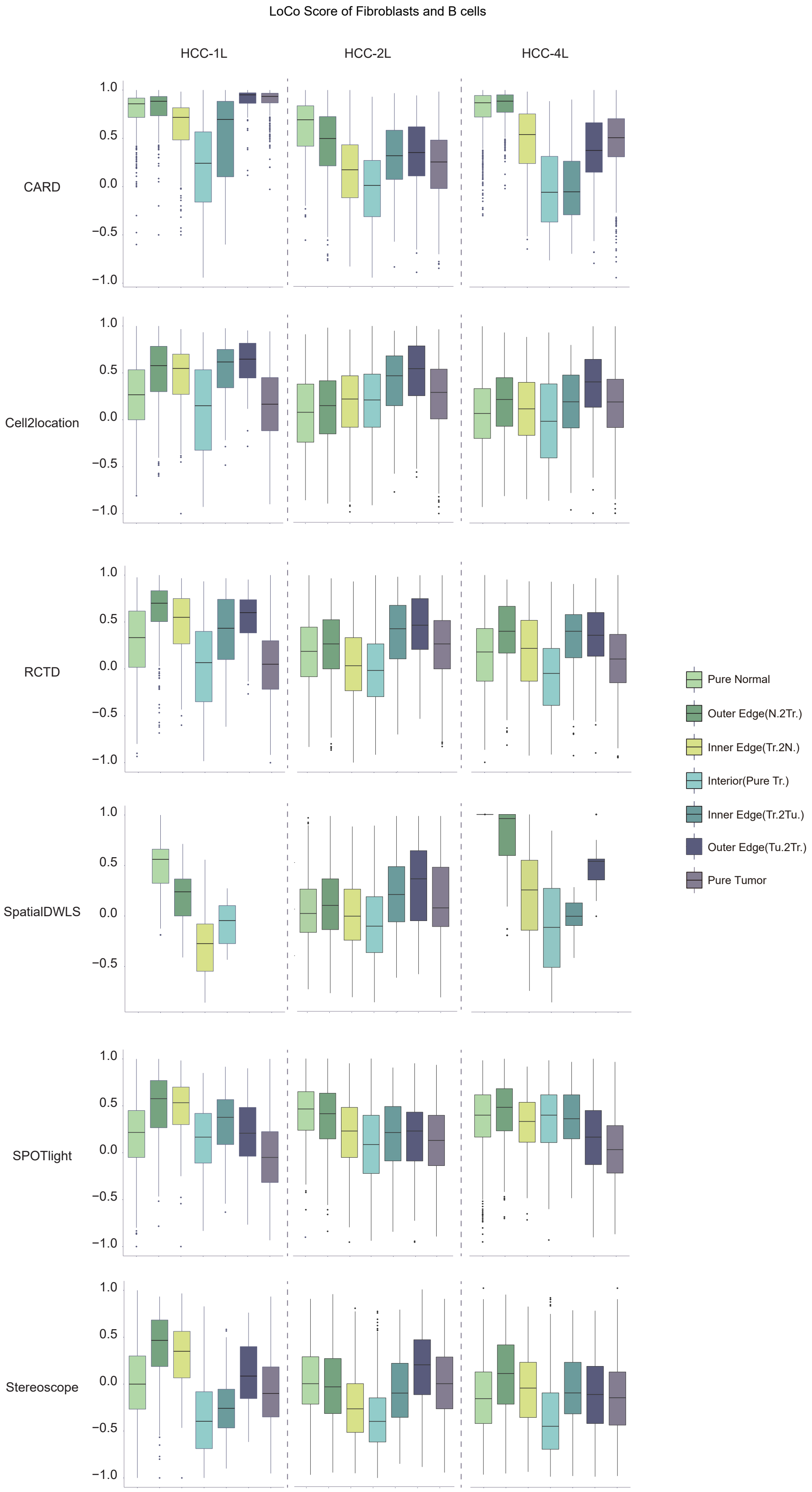

LoCo Score of Fibroblasts and B cells
HCC-1L
HCC-2L
HCC-4L
CARD
Cell2location
RCTD
SpatialDWLS
SPOTlight
Stereoscope
1.0
0.5
0.0
−0.5
−1.0
Pure Normal
Outer Edge(N.2Tr.)
Inner Edge(Tr.2N.)
Interior(Pure Tr.)
Inner Edge(Tr.2Tu.)
Outer Edge(Tu.2Tr.)
Pure Tumor

Supplementary Fig. 27 LoCo Scores of Fibroblasts and B cells by all algorithms in detailed regions of 3 patients.

The LoCo scores of Fibroblasts and B cells in detailed regions calculated by all algorithms for 3 patients are presented (n = 781; 314; 182; 120; 78; 80; 1,199; 330; 727; 790; 865; 275; 295; 1,386; 877; 570; 236; 175; 108; 215; 1,926 spots from left to right boxes for each algorithms, respectively). The legend (region annotation) is same as Fig. 5e. Each box plot ranges from the first and third quartiles with the median as the horizontal line, while whiskers represent 1.5 times the interquartile range from the lower and upper bounds of the box. Source data are provided as a Source Data file.

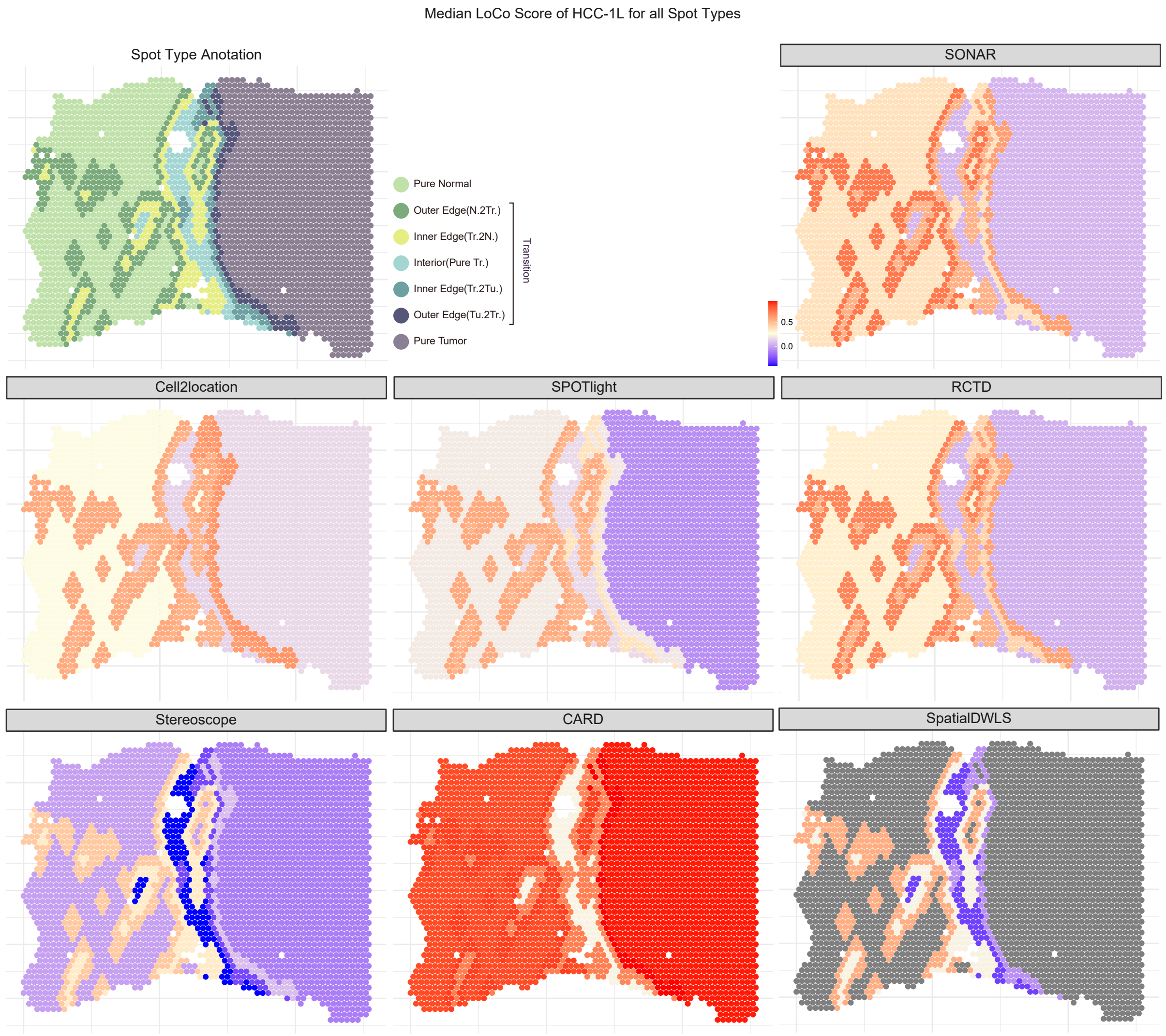

Supplementary Fig. 28 Median LoCo scores of Fibroblasts and B cells by Spot Type for in HCC-1L.

The top, left figure show the Spot Type annotation (same as Figure 5e), different colors represent different Spot Type. Specifically, the spots are stratified according to the regional composition of their neighboring set into 7 types: Pure Normal, Outer edge to Transition region (Normal or Tumor side), Inner edge to Transition region (Normal or Tumor side), Interior Transition region, and Pure Tumor region. Other figures show the median (by Spot Type) LoCo scores of Fibroblasts and B cells on the spatial spots for SONAR, Cell2location, SPOTlight, RCTD, Stereoscope, CARD and SpatialDWLS. The color bar represents the value of LoCo scores. Source data are provided as a Source Data file.

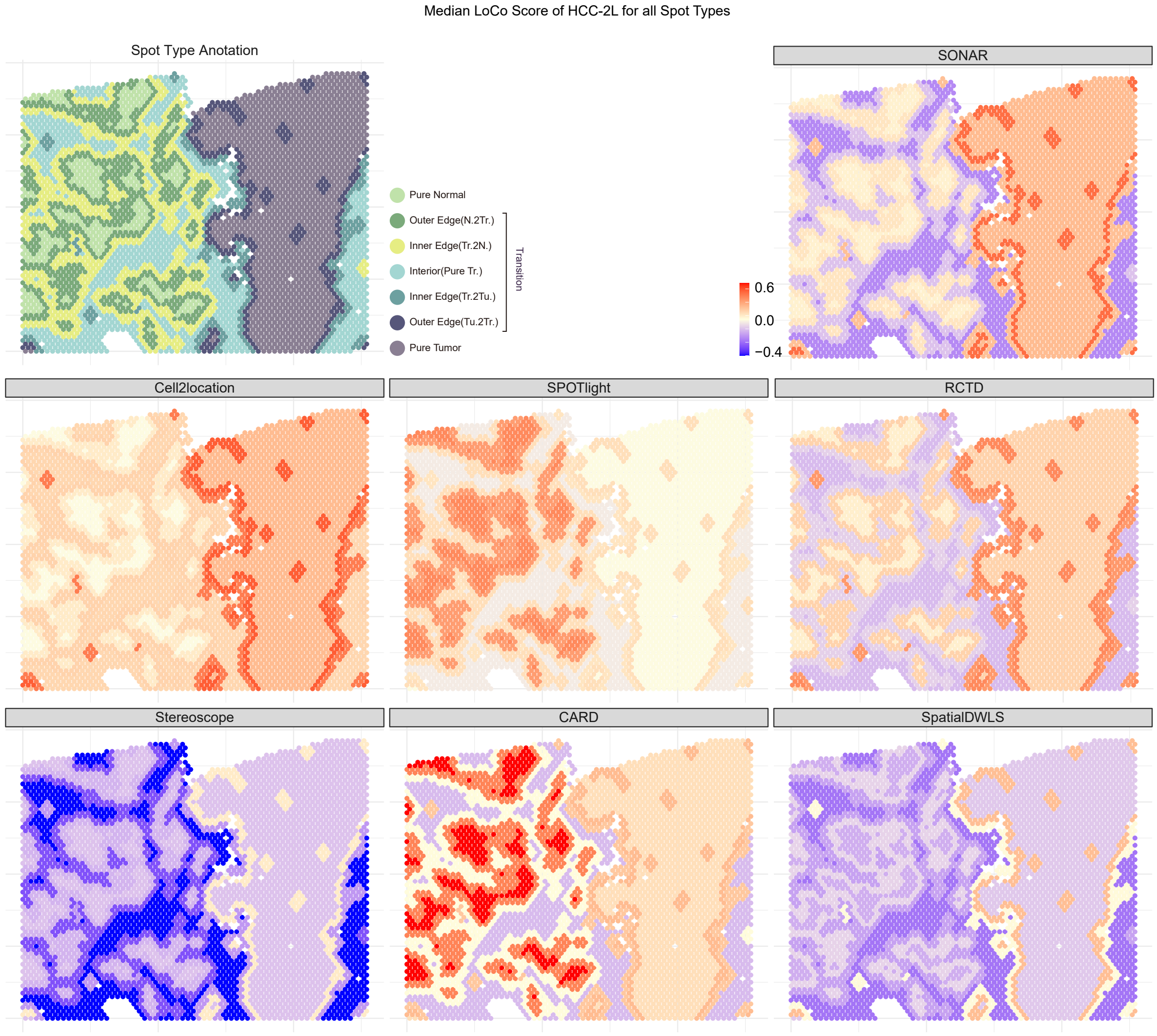

Supplementary Fig. 29 Median LoCo scores of Fibroblasts and B cells by Spot Type for in HCC-2L.

The top, left figure show the Spot Type annotation (same as Figure 5e), different colors represent different Spot Type. Specifically, the spots are stratified according to the regional composition of their neighboring set into 7 types: Pure Normal, Outer edge to Transition region (Normal or Tumor side), Inner edge to Transition region (Normal or Tumor side), Interior Transition region, and Pure Tumor region. Other figures show the median (by Spot Type) LoCo scores of Fibroblasts and B cells on the spatial spots for SONAR, Cell2location, SPOTlight, RCTD, Stereoscope, CARD and SpatialDWLS. The color bar represents the value of LoCo scores. Source data are provided as a Source Data file.

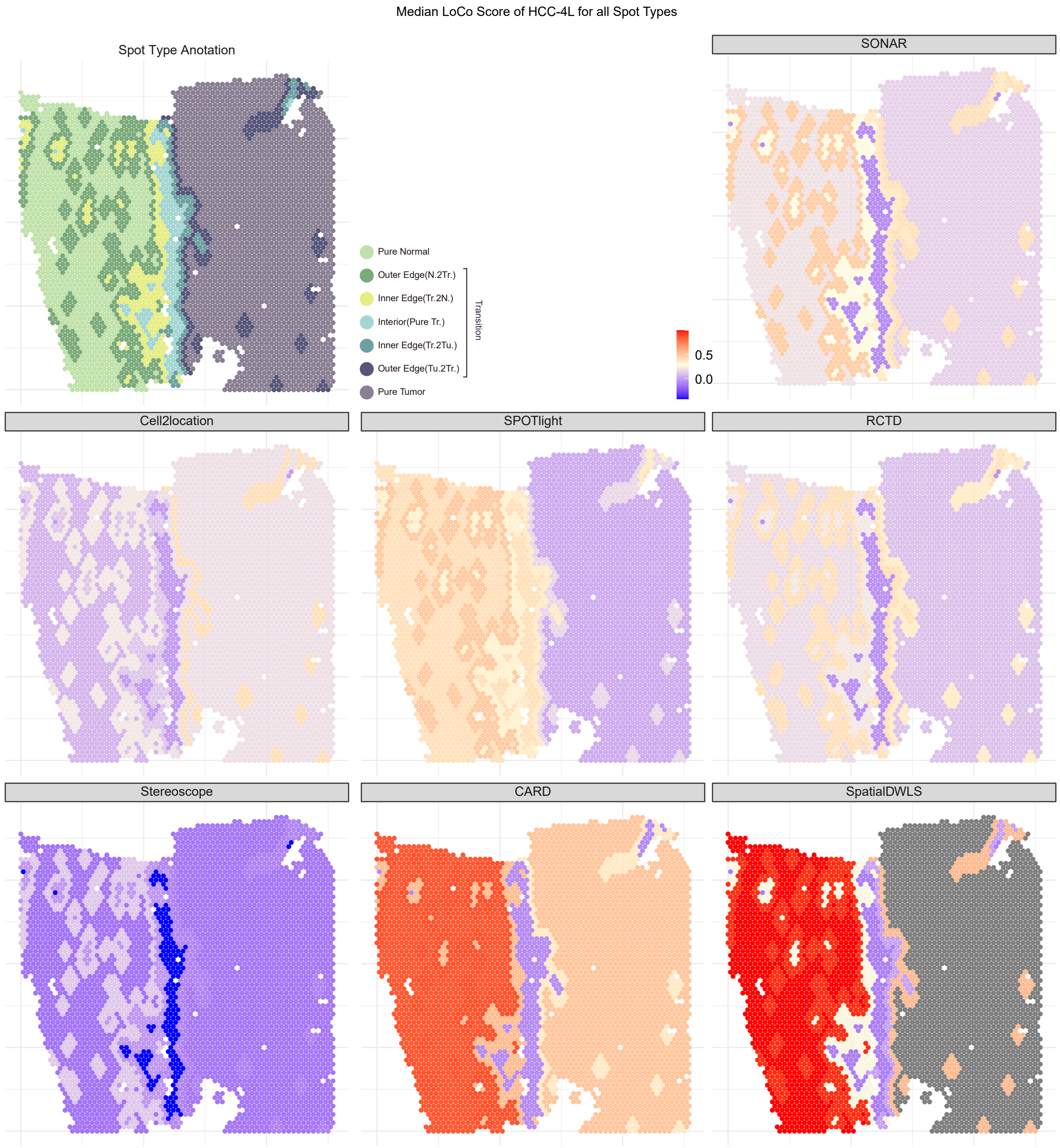

Supplementary Fig. 30 Median LoCo scores of Fibroblasts and B cells by Spot Type for in HCC-4L.

The top, left figure show the Spot Type annotation (same as Figure 5e), different colors represent different Spot Type. Specifically, the spots are stratified according to the regional composition of their neighboring set into 7 types: Pure Normal, Outer edge to Transition region (Normal or Tumor side), Inner edge to Transition region (Normal or Tumor side), Interior Transition region, and Pure Tumor region. Other figures show the median (by Spot Type) LoCo scores of Fibroblasts and B cells on the spatial spots for SONAR, Cell2location, SPOTlight, RCTD, Stereoscope, CARD and SpatialDWLS. The color bar represents the value of LoCo scores. Source data are provided as a Source Data file.

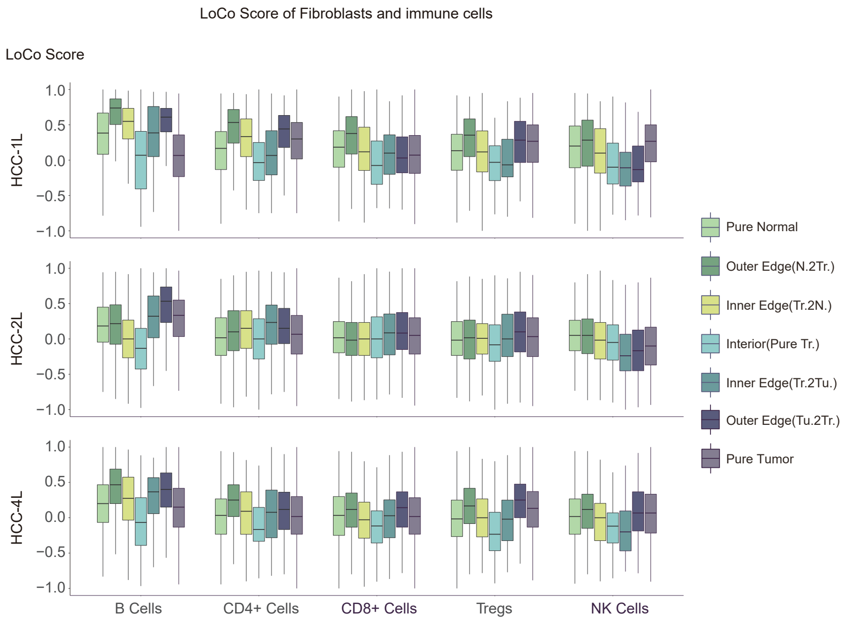

Supplementary Fig. 31 LoCo Scores of Fibroblasts and immune cells in detailed regions of 3 patients.
The LoCo scores of Fibroblasts and all immune cells in detailed regions calculated by SONAR for 3 patients are presented (n = 781; 314; 182; 120; 78; 80; 1,199; 330; 727; 790; 865; 275; 295; 1,386; 877; 570; 236; 175; 108; 215; 1,926 spots from Pure Normal to Pure Tumor in HCC-1L, HCC-2L, HCC-4L, respectively). The legend (region annotation) is same as Fig. 5e. Each box plot ranges from the first and third quartiles with the median as the horizontal line, while whiskers represent 1.5 times the interquartile range from the lower and upper bounds of the box. Source data are provided as a Source Data file.

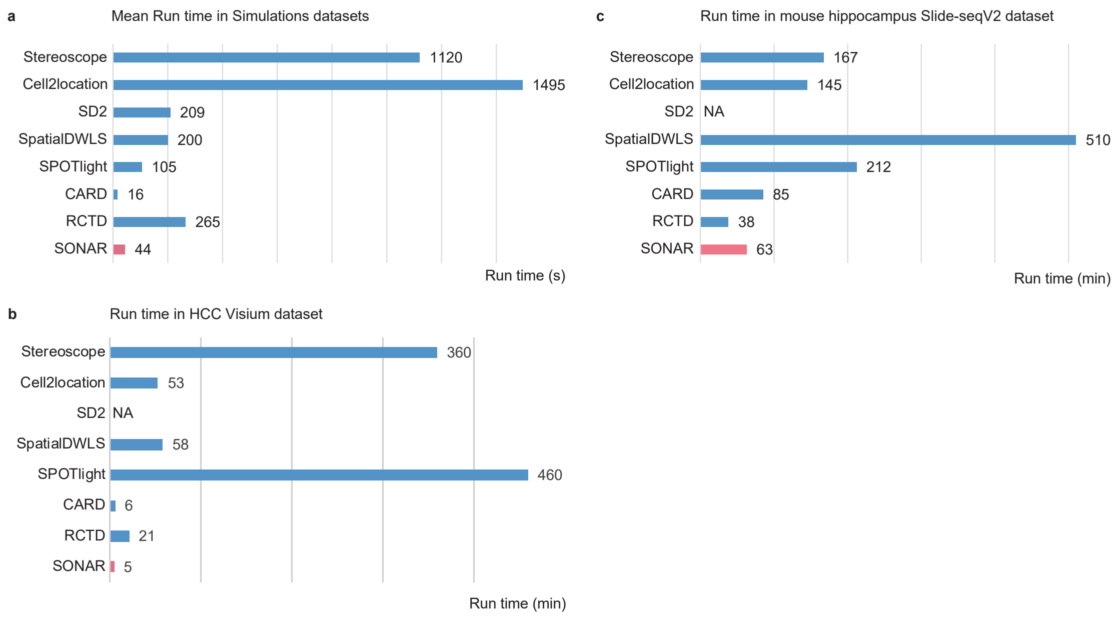

Supplementary Fig. 32 Computational time of comparing algorithms.
a, the bar plot shows the computational time for the simulation datasets which contains 800 spots and 2,845 genes. **b**, the computational time for a liver cancer(HCC) Visium dataset, which contains 2,791 spots and 17,735 genes. **c**, the computational time for a large-scale Slide-seqV2 dataset, which contains 41,795 spots and 5,093 genes. The computation time was in units of seconds in **a**, and in units of minutes **b** and **c**.

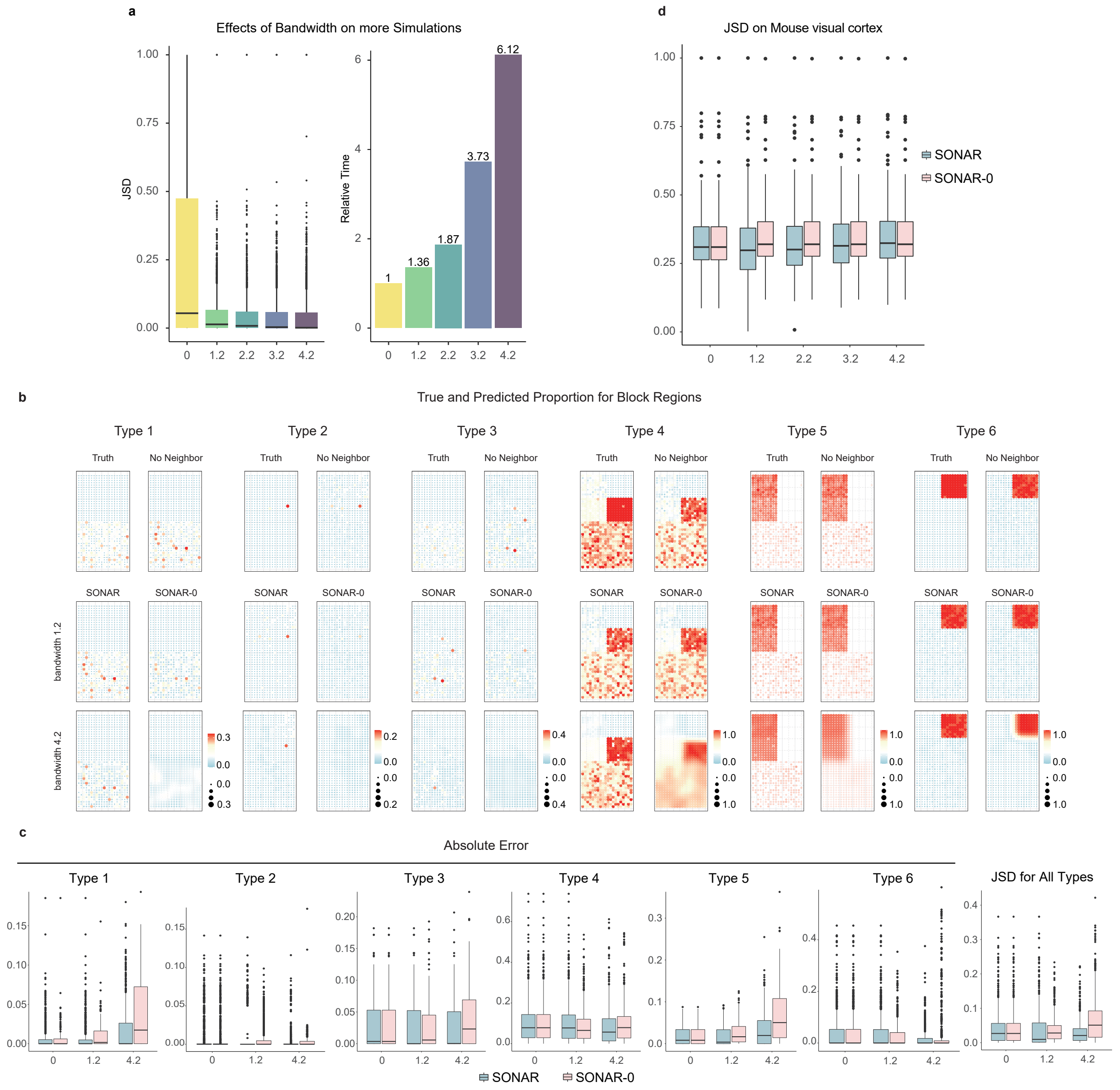

Supplementary Fig. 33 Effect of bandwidth on Simulation data and Mouse visual cortex data.

a, Effects of different bandwidths on accuracy and speed in 4 simulation datasets. The bandwidth with no neighbor information is denoted as 0. The distance for the nearest pair of spots is denoted as 1. The boxplot shows the JSD for each spot under the different bandwidths (0, 1.2, 2.2, 3.2, 4.2) in the block and background patterns (4 datasets which each contains 800 spots). The barplot represents the computation times of different bandwidths relative to bandwidth 0. The colors represent different bandwidths. **b**, Spatial scatter plots of the true and predicted proportions for each cell type in the Block pattern simulation under different bandwidths. The first row shows the true proportions (left) and the predictions without neighbor information (right). The second and third rows show the predictions by SONAR and SONAR-0 with bandwidths of 1.2 and 4.2, respectively. The color and size of the scatters indicate the proportions for each spot. **c**, Boxplots of the absolute errors for each

cell type under different bandwidths (n = 800 spots). The colors represent SONAR and SONAR-0. The absolute error is calculated by the absolute difference between the true and predicted proportions for each spot. The rightmost column summarizes the Jensen-Shannon divergence (JSD) for all spots and all cell types (n = 800 spots). **d**, Boxplots of the JSD for the mouse brain dataset under different bandwidths, 0 to 4.2 (n = 189 spots). The colors represent SONAR and SONAR-0. Each box in **c** and **d** plot ranges from the first and third quartiles with the median as the horizontal line, while whiskers represent 1.5 times the interquartile range from the lower and upper bounds of the box. Source data are provided as a Source Data file.

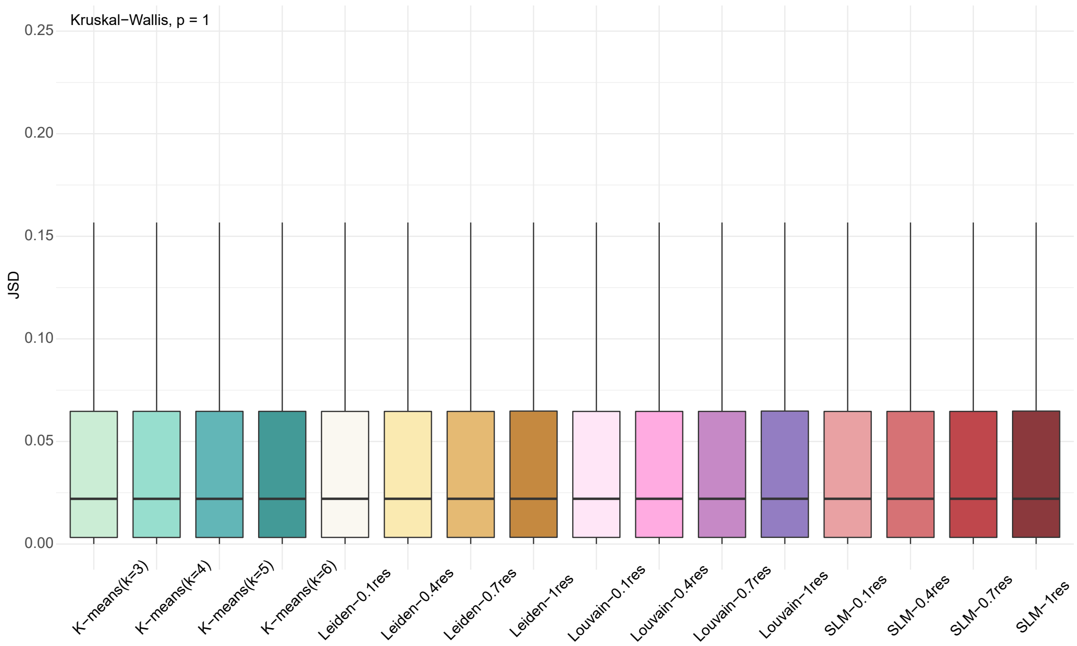

Supplementary Fig. 34 Robust on different clustering algorithms and clustering parameters.

The boxplot shows the JSD for each spot under the K-means (k=3, 4, 5, 6, from left to right), Leiden, Louvain and SLM clustering algorithms with different resolution parameters (0.1, 0.4, 0.7, 1, from left to right) (n = 800 spots). Each box plot ranges from the first and third quartiles with the median as the horizontal line, while whiskers represent 1.5 times the interquartile range from the lower and upper bounds of the box. We used the Kruskal-Wallis test to assess the differences among different conditions, p-value = 1. Source data are provided as a Source Data file.

References

[1] Fotheringham, A. S., Brunsdon, C. & Charlton, M. *Geographically Weighted Regression: The Analysis of Spatially Varying Relationships***13** (John Wiley & Sons, 2002).

[2] Fotheringham, A. S., Yang, W. & Kang, W. Multiscale Geographically Weighted Regression (MGWR). *Annals of the American Association of Geographers* **107**, 1247–1265 (2017).

[3] Wang, X. *et al.* Three-dimensional intact-tissue sequencing of single-cell transcriptional states. *Science* **361**, eaat5691. https://doi.org/10.1126/science.aat5691 (2018).

[4] Li, B. *et al.* Benchmarking spatial and single-cell transcriptomics integration methods for transcript distribution prediction and cell type deconvolution. *Nature Methods* **19**, 662–670. https://doi.org/10.1038/s41592-022-01480-9 (2022).

[5] Chen, J. *et al.* A comprehensive comparison on cell-type composition inference for spatial transcriptomics data. *Briefings in Bioinformatics* **23**, bbac245. https://doi.org/10.1093/bib/bbac245 (2022).

[6] Cable, D.M., Murray, E., Zou, L.S. *et al.* Robust decomposition of cell type mixtures in spatial transcriptomics. *Nature Biotechnology* **40**, 517–526. https://doi.org/10.1038/s41587-021-00830-w (2022).

[7] Ma, Y., Zhou, X. Spatially informed cell-type deconvolution for spatial transcriptomics. *Nature Biotechnology* **40**, 1349–1359. https://doi.org/10.1038/s41587-022-01273-7 (2022).

[8] Dong, R. & Yuan, GC. SpatialDWLS: accurate deconvolution of spatial transcriptomic data. *Genome Biology* **22**, 145. https://doi.org/10.1186/s13059-021-02362-7 (2021).

[9] Elosua-Bayes, M. *et al.* SPOTlight: seeded NMF regression to deconvolute spatial transcriptomics spots with single-cell transcriptomes. *Nucleic Acids Research* **49**, e50. https://doi.org/10.1093/nar/gkab043 (2021).

[10] Andersson, A. *et al.* Single-cell and spatial transcriptomics enables probabilistic inference of cell type topography. *Communications Biology* **3**, 565. https://doi.org/10.1038/s42003-020-01247-y (2020).

[11] Kleshchevnikov, V. *et al.* Cell2location maps fine-grained cell types in spatial transcriptomics. *Nature Biotechnology* **40**, 661–671. https://doi.org/10.1038/s41587-021-01139-4 (2022).

[12] Li, H., Li, H., Zhou, J. & Gao, X. SD2: spatially resolved transcriptomics deconvolution through integration of dropout and spatial information. *Bioinformatics* **38**, 4878–4884. https://doi.org/10.1093/bioinformatics/btac605 (2022).

Supplementary Table 1: Markers list		
**Cell types**	**Genes**	
Astrocyte	*Slc1a3*	*Aqp4*
CA1	*Wfs1*	
CA3	*Hs3st4*	
CR	*Trp73*	
Choroid	*Folr1*	
Dentate	*Prox1*	*C1ql2*
Endothelial Stalk	*Cldn5*	
Endothelial Tip	*Nid1*	
Ependymal	*Ccdc153*	
Microglia Macrophages	*P2ry12*	
Mural	*Rgs5*	*Acta2*
Neuron.Slc17a6	*Slc17a6*	
Oligodendrocyte	*Mbp*	
Polydendrocyte	*Pdgfra*	

Supplementary Table 2: Correlations between predicted proportion and cell type markers in Slide-seqV2 dataset

	SONAR	RCTD	CARD	SpatialDWLS	SPOTlight	Cell2location	Stereoscope
Astrocyte	0.230	**0.248**	0.212	0.155	0.233	0.196	0.241
CA1	0.291	0.171	0.186	**0.336**	0.111	0.217	0.216
CA3	0.165	0.162	0.221	0.241	0.199	**0.251**	0.145
Cajal Retzius	0.024	**0.046**	0.033	-0.003	0.031	0.028	0.014
Choroid	**0.463**	0.146	0.138	0.457	0.122	0.145	0.146
Denate	0.226	0.202	0.161	**0.335**	0.194	0.214	0.230
Endothelial Stalk	**0.134**	0.105	0.084	0.021	0.094	0.101	0.107
Endothelial Tip	**0.111**	0.058	0.042	0.089	0.058	0.051	0.035
Ependymal	**0.434**	0.225	0.209	0.261	0.183	0.211	0.226
Microglia Macrophages	**0.258**	0.188	0.161	0.161	0.166	0.173	0.190
Mural	0.193	**0.221**	0.069	0.051	0.137	0.179	0.216
Neuron.Slc17a6	0.072	0.150	0.157	0.011	0.091	**0.201**	-0.032
Oligodendrocyte	0.473	**0.573**	0.379	0.365	0.430	0.515	0.532
Polydendrocyte	0.180	0.139	0.097	0.086	0.125	0.113	0.123
AVERAGE	**0.232**	0.188	0.153	0.183	0.155	0.186	0.171

The value indicates the Spearman correlation of predicted proportion to cell type marker for all algorithms. Higher values indicate better performance. The top one correlations for each cell type are shown in bold.

Supplementary Table 3: Markers list		
**Cell types**	**Genes**	
Acinar cells	*PRSS1*	*PRSS2*
Cancer clone A	*TM4SF1*	
Cancer clone B	*S100A4*	*IFI27*
Ductal antigen-presenting	*C4A*	
Ductal centroacinar	*SERPINA1*	
Ductal high hypoxic	*CLDN4*	
Ductal terminal	*TFF2*	
Endocrine cells	*PCSK1N*	
Endothelial cells	*EMP1*	
Fibroblasts	*LGALS1*	
Macrophages A	*CTSL*	
Macrophages B	*ATP6V1B2*	
Mast cells	*MS4A2*	
mDCs A	*ALOX5AP*	*FCER1G*
mDCs B	*APOE*	
Monocytes	*S100A9*	
pDCs	*MAPKAPK2*	
RBCs	*HBA1*	
T cells and NK cells	*LCP1*	
Tuft cells	*AZGP1*	

Supplementary Table 4: Global correlations between predicted proportion and cell type specific marker genes								
	SONAR	RCTD	CARD	SpatialDWLS	SPOTlight	Cell2location	Stereoscope	SD2
Acinar cells	0.528	0.528	0.415	0.550	0.385	0.500	0.504	**0.555**
Cancer clone A	0.405	0.387	**0.423**	0.354	0.409	0.296	0.378	0.313
Cancer clone B	0.322	0.318	0.264	0.257	0.317	0.282	0.280	**0.351**
Ductal antigen-presenting	0.226	-0.009	**0.410**	0.115	0.221	0.366	0.022	0.133
Ductal centroacinar	**0.500**	0.456	0.451	0.457	0.435	0.391	0.278	0.375
Ductal high hypoxic	0.107	-0.023	0.162	-0.005	**0.222**	0.094	0.057	-0.182
Ductal terminal	**0.287**	0.258	0.268	0.092	0.222	0.279	0.275	0.158
Endocrine cells	0.212	0.218	0.217	-0.021	0.080	**0.236**	0.182	NA
Endothelial cells	**0.111**	0.080	0.097	0.110	0.079	0.069	0.092	0.030
Fibroblasts	0.409	0.434	0.376	0.390	0.403	0.403	**0.460**	0.204
Macrophages A	0.137	0.053	0.130	0.064	**0.187**	0.118	0.015	-0.009
Macrophages B	0.183	0.060	0.129	-0.050	0.107	**0.262**	0.169	0.051
Mast cells	**0.153**	0.121	0.094	NA	0.083	0.114	0.126	-0.066
mDCs A	**0.225**	0.175	0.224	0.009	0.212	0.071	0.084	NA
mDCs B	**0.280**	0.258	0.277	0.079	-0.138	0.018	0.280	0.079
Monocytes	0.213	0.318	**0.325**	-0.028	0.152	0.126	0.324	0.035
pDCs	**0.220**	0.170	0.174	-0.005	0.134	0.070	0.048	NA
RBCs	**0.286**	0.184	0.200	NA	0.103	-0.006	0.131	0.098
T cells and NK cells	**0.145**	0.111	0.112	-0.021	0.080	0.089	0.091	-0.014
Tuft cells	0.251	0.215	**0.306**	-0.004	0.245	0.248	0.189	0.206
AVERAGE	**0.260**	0.216	0.253	0.130	0.197	0.201	0.199	0.136

The value indicates the Spearman correlation of predicted proportion to cell type marker for all algorithms. Higher values indicate better performance. The top one correlations for each cell type are shown in bold.

Supplementary Table 5: Regional correlations between predicted proportion and cell type specific marker genes

		SONAR	RCTD	CARD	SpatialDWLS	SPOTlight	Cell2location	Stereoscope	SD2
Pancreatic region	Acinar cells	0.814	0.807	0.770	0.806	0.745	0.655	**0.871**	0.773
	Endocrine cells	**0.334**	0.278	0.288	-0.025	0.138	0.313	0.265	NA
	mDCs A	0.340	0.250	0.329	-0.081	**0.380**	0.242	0.064	NA
Cancer region	Cancer clone A	0.506	0.491	**0.576**	0.269	0.503	0.355	0.470	0.281
	Cancer clone B	0.360	0.413	0.312	0.294	0.331	0.414	0.414	**0.464**
	Fibroblasts	0.444	0.425	0.428	0.409	0.418	0.416	**0.448**	0.186
Duct region	Ductal terminal	0.348	0.184	**0.411**	0.176	0.295	0.186	0.322	0.274
	Ductal centroacinar	**0.697**	0.578	0.612	0.519	0.323	0.549	0.390	0.445
Stroma region	Acinar cells	0.522	0.512	0.430	0.502	0.334	0.464	0.488	**0.571**
	Duct centroacinar	0.413	0.331	**0.424**	0.334	0.316	0.293	0.169	0.223
	mDCs B	0.298	0.258	0.239	0.121	-0.110	-0.171	0.275	0.198
AVERAGE		**0.461**	0.412	0.438	0.302	0.334	0.338	0.380	0.379

The value indicates the Spearman correlation of predicted proportion to cell type marker for all algorithms. Higher values indicate better performance. The top one correlations for each cell type are shown in bold.

Supplementary Table 6: Median of LoCo scores of Fibroblasts and B Cells by Spot Type

Method	Patient	Trend	Median of Loco scores by Spot Type						
			Pure Normal	Outer Edge (N.2Tr.)	Inner Edge (Tr.2N.)	Interior	Inner Edge (Tr.2Tu.)	Outer Edge (Tu.2Tr.)	Pure Tumor
SONAR	HCC-1L	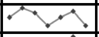	0.383	**0.738**	0.549	0.069	0.386	**0.608**	0.067
	HCC-2L	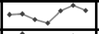	0.183	0.217	0.000	-0.133	0.321	**0.533**	**0.333**
	HCC-4L	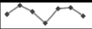	0.200	**0.467**	0.275	-0.067	0.367	**0.400**	0.150
Cell2location	HCC-1L	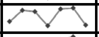	0.267	0.577	0.548	0.150	**0.617**	**0.646**	0.167
	HCC-2L	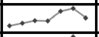	0.133	0.200	0.267	0.257	**0.500**	**0.571**	0.333
	HCC-4L	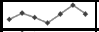	0.067	**0.217**	0.117	-0.017	0.192	**0.405**	0.190
SPOTlight	HCC-1L	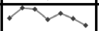	0.217	**0.576**	**0.533**	0.167	0.377	0.208	-0.050
	HCC-2L	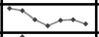	**0.467**	**0.417**	0.233	0.095	0.217	0.233	0.133
	HCC-4L	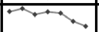	**0.400**	**0.483**	0.333	**0.400**	0.362	0.167	0.033
RCTD	HCC-1L	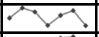	0.333	**0.700**	0.550	0.067	0.433	**0.600**	0.050
	HCC-2L	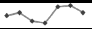	0.187	0.267	0.033	-0.017	**0.429**	**0.467**	0.267
	HCC-4L	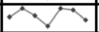	0.179	**0.400**	0.217	-0.050	**0.400**	0.357	0.104
Stereoscope	HCC-1L	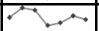	0.000	**0.467**	**0.350**	-0.396	-0.258	0.086	-0.100
	HCC-2L	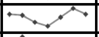	**0.000**	-0.033	-0.267	-0.400	-0.100	**0.200**	**0.000**
	HCC-4L	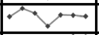	-0.143	**0.117**	**-0.033**	-0.429	-0.083	-0.100	-0.133
CARD	HCC-1L	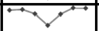	0.857	0.883	0.717	0.242	0.695	**0.950**	**0.933**
	HCC-2L	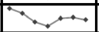	**0.683**	**0.483**	0.150	-0.017	0.300	0.333	0.233
	HCC-4L	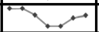	**0.867**	**0.883**	0.533	-0.071	-0.067	0.367	0.500
SpatialDWLS	HCC-1L	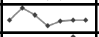	NA	**0.561**	**0.240**	-0.274	-0.046	NA	NA
	HCC-2L	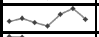	-0.046	0.040	-0.075	-0.183	**0.157**	**0.325**	0.014
	HCC-4L	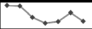	**1.000**	**0.959**	0.259	-0.109	0.000	0.540	NA

The table shows the median LoCo Scores by spot type for each method. The top two correlations correspond to two peaks for each patient are shown in bold.

Supplementary Table 7: Moran's I for All cell types in Simulation data						
	Cell Type 1	Cell Type 2	Cell Type 3	Cell Type 4	Cell Type 5	Cell Type 6
bandwidth 1.2	0.343	-0.003	0.280	0.844	0.838	**0.931**
bandwidth 2.2	0.329	-0.003	0.292	0.809	0.803	**0.884**
bandwidth 3.2	0.319	-0.003	0.285	0.742	0.736	**0.801**
bandwidth 4.2	0.314	-0.003	0.279	0.702	0.697	**0.751**
The value indicates the Moran's I for all cell types. The top one value for each bandwidth are shown in bold.						
