# Supplementary material for: SONAR enables cell type deconvolution with spatially weighted Poisson-Gamma model for spatial transcriptomics
Source: Nat Commun. 2023 Aug 7;14:4727. doi: 10.1038/s41467-023-40458-9 (PMC10406862; doi:10.1038/s41467-023-40458-9)
Supplement: Supplementary file 3 — Reporting Summary [file 41467_2023_40458_MOESM3_ESM.pdf]

nature portfolio

Corresponding author(s): Liang Ma
Last updated by author(s): Jul 11, 2023

Reporting Summary

Nature Portfolio wishes to improve the reproducibility of the work that we publish. This form provides structure for consistency and transparency in reporting. For further information on Nature Portfolio policies, see our Editorial Policies and the Editorial Policy Checklist.

Statistics

For all statistical analyses, confirm that the following items are present in the figure legend, table legend, main text, or Methods section.

n/a	Confirmed	
☐	☒	The exact sample size (*n*) for each experimental group/condition, given as a discrete number and unit of measurement
☐	☒	A statement on whether measurements were taken from distinct samples or whether the same sample was measured repeatedly
☐	☒	The statistical test(s) used AND whether they are one- or two-sided<br>*Only common tests should be described solely by name; describe more complex techniques in the Methods section.*
☐	☒	A description of all covariates tested
☐	☒	A description of any assumptions or corrections, such as tests of normality and adjustment for multiple comparisons
☐	☒	A full description of the statistical parameters including central tendency (e.g. means) or other basic estimates (e.g. regression coefficient) AND variation (e.g. standard deviation) or associated estimates of uncertainty (e.g. confidence intervals)
☐	☒	For null hypothesis testing, the test statistic (e.g. *F*, *t*, *r*) with confidence intervals, effect sizes, degrees of freedom and *P* value noted<br>*Give P values as exact values whenever suitable.*
☒	☐	For Bayesian analysis, information on the choice of priors and Markov chain Monte Carlo settings
☐	☒	For hierarchical and complex designs, identification of the appropriate level for tests and full reporting of outcomes
☐	☒	Estimates of effect sizes (e.g. Cohen's *d*, Pearson's *r*), indicating how they were calculated

Our web collection on statistics for biologists contains articles on many of the points above.

Software and code

Policy information about availability of computer code

Data collection	No software was used for data collection.
Data analysis	We used the newly developed R package SONAR for data analysis, with the open-source code and dependency packages information available at GitHub: [https://github.com/lzygenomics/SONAR]. For comparative analysis, we used the following softwares: CARD (R package, version 1.0), RCTD (R package, version 1.2.0), SPOTlight (R package, version 0.1.7), SpatialDWLS (R package, version 1.1.1), Cell2location (Python package, version 0.1), Stereoscope (Python package, version 0.3.1), SD2 (SD2 is a Python script that is not yet a package, there is no version information, and the author was last seen updating the script on Sep 22, 2022, as of this writing). For benchmarking pre-clustering method and resolution, we used Seurat (R package, version 4.0.3) to perform Louvain, SLM and Leiden clustering, and used Giotto (R package, version 1.1.1) to perform K-means clustering. For gridding high resolution heart data, we also used Giotto (R package, version 1.1.1).

For manuscripts utilizing custom algorithms or software that are central to the research but not yet described in published literature, software must be made available to editors and reviewers. We strongly encourage code deposition in a community repository (e.g. GitHub). See the Nature Portfolio guidelines for submitting code & software for further information.

Data

Policy information about availability of data

All manuscripts must include a data availability statement. This statement should provide the following information, where applicable:

- Accession codes, unique identifiers, or web links for publicly available datasets
- A description of any restrictions on data availability
- For clinical datasets or third party data, please ensure that the statement adheres to our policy

This study made use of publicly available datasets. The data were acquired from the following websites or accession numbers.
For simulation materials, we used the Annotated PBMC scRNA-seq data, which are publicly available at [https://github.com/MarcElosua/SPOTlight_deconvolution_analysis/tree/master/analysis/tool_benchmarking]. This data is original from the paper (Mereu, E et al. 2020).
For gridded mouse visual cortex spatial transcriptomic data, annotated scRNA-seq reference, and the results of other algorithms on this dataset are provided by the platform (Li, B et al. 2022) at [https://github.com/QuKunLab/SpatialBenchmarking/tree/main/FigureData/Figure4/Dataset10_STARmap]. These raw data are derived from the publications (Wang, X et al. 2018; Tasic, B et al. 2018).
For gridded human heart data and annotated scRNA-seq reference are publicly available at [https://github.com/JiawenChenn/St-review/tree/main/processed_data/heart/ISS]. These raw data are sourced from (Asp, M et al. 2019).
For PDAC spatial datasets and the paired reference are available at [https:// www.ncbi.nlm.nih.gov/geo/query/acc.cgi?acc=GSE111672]. We use the PDAC-A sample for analysis.
For liver data, the accession number for the liver cancer spatial data deposited in Genome Sequence Archive (GSA) is HRA000437, we use the HCC-1L, HCC-2L, HCC-3L, HCC-4L samples. The scRNA-seq reference in liver cancer analysis is available at Mendeley data [https://doi.org/10.17632/6wmzcskt6k.1].
The mouse hippocampus Slide-seqV2 dataset and annotated scRNA-seq data are available at https://singlecell.broadinstitute.org/single_cell/study/SCP948/robust-decomposition-of-cell-type-mixtures-in-spatial-transcriptomics.

Research involving human participants, their data, or biological material

Policy information about studies with human participants or human data. See also policy information about sex, gender (identity/presentation), and sexual orientation and race, ethnicity and racism.

Reporting on sex and gender	*Use the terms sex (biological attribute) and gender (shaped by social and cultural circumstances) carefully in order to avoid confusing both terms. Indicate if findings apply to only one sex or gender; describe whether sex and gender were considered in study design; whether sex and/or gender was determined based on self-reporting or assigned and methods used. Provide in the source data disaggregated sex and gender data, where this information has been collected, and if consent has been obtained for sharing of individual-level data; provide overall numbers in this Reporting Summary. Please state if this information has not been collected. Report sex- and gender-based analyses where performed, justify reasons for lack of sex- and gender-based analysis.*
Reporting on race, ethnicity, or other socially relevant groupings	*Please specify the socially constructed or socially relevant categorization variable(s) used in your manuscript and explain why they were used. Please note that such variables should not be used as proxies for other socially constructed/relevant variables (for example, race or ethnicity should not be used as a proxy for socioeconomic status). Provide clear definitions of the relevant terms used, how they were provided (by the participants/respondents, the researchers, or third parties), and the method(s) used to classify people into the different categories (e.g. self-report, census or administrative data, social media data, etc.) Please provide details about how you controlled for confounding variables in your analyses.*
Population characteristics	*Describe the covariate-relevant population characteristics of the human research participants (e.g. age, genotypic information, past and current diagnosis and treatment categories). If you filled out the behavioural & social sciences study design questions and have nothing to add here, write "See above."*
Recruitment	*Describe how participants were recruited. Outline any potential self-selection bias or other biases that may be present and how these are likely to impact results.*
Ethics oversight	*Identify the organization(s) that approved the study protocol.*

Note that full information on the approval of the study protocol must also be provided in the manuscript.

Field-specific reporting

Please select the one below that is the best fit for your research. If you are not sure, read the appropriate sections before making your selection.

☒ Life sciences ☐ Behavioural & social sciences ☐ Ecological, evolutionary & environmental sciences

For a reference copy of the document with all sections, see nature.com/documents/nr-reporting-summary-flat.pdf

Life sciences study design

All studies must disclose on these points even when the disclosure is negative.

Sample size	We evaluated SONAR across 8 spatial transcriptomics datasets in real data applications, including:<br>Mouse cortex data, with 1549 spots and a scRNA-seq reference of 14249 cells;

Mouse hippocampus Slide-seqV2 data, with 41795 spots and a scRNA-seq reference of 15095 cells;
Human heart data, with 17444 spots and a scRNA-seq reference of 3254 cells;
PDAC data, with 428 spots and a scRNA-seq reference of 1926 cells;
Four liver datasets (HCC-1L, HCC-2L, HCC-3L, HCC-4L), with 2791, 4672, 4758, and 4113 spots respectively, and a common scRNA-seq reference of 73589 cells;

For real data analysis, we did not use any statistical methods to pre-determine sample sizes, but our sample sizes (8 datasets) are similar to those reported by other compared deconvolution algorithms. Moreover, within each dataset, we used all spots or cells without the need to determine sample size.

For simulation datasets, we used:
80 datasets for Homo-Area scheme, with 5 replications for each scenario in Homo-Area scheme;
95 datasets for Compo-Area scheme, with 5 replications for each scenario in Compo-Area scheme;

The number of samples (80 and 95) were determined by multiplying the number of replications by the number of scenarios for each case.

Data exclusions	No data was excluded from this study.
Replication	To evaluate the performance of SONAR and other deconvolution methods, we performed simulations and each simulation was replicated for 5 times to make the results stable.
Randomization	For real data, Randomization is not relevant to this study because each samples or slide was analyzed separately, and in each sample, we used all spots. For simulation, different replications for each scenarios were generated randomly.
Blinding	In the comparison, all the methods were blinded to the ground truth of the spatial transcriptomics data. The outputs from the methods were then compared to the ground truth available in the respective datasets.

Reporting for specific materials, systems and methods

We require information from authors about some types of materials, experimental systems and methods used in many studies. Here, indicate whether each material, system or method listed is relevant to your study. If you are not sure if a list item applies to your research, read the appropriate section before selecting a response.

Materials & experimental systems

n/a	Involved in the study
☒	☐ Antibodies
☒	☐ Eukaryotic cell lines
☒	☐ Palaeontology and archaeology
☒	☐ Animals and other organisms
☒	☐ Clinical data
☒	☐ Dual use research of concern
☒	☐ Plants

Methods

n/a	Involved in the study
☒	☐ ChIP-seq
☒	☐ Flow cytometry
☒	☐ MRI-based neuroimaging
